# Tagging enhances histochemical and biochemical detection of Ran Binding Protein 9 *in vivo* and reveals its interaction with Nucleolin

**DOI:** 10.1038/s41598-020-64047-8

**Published:** 2020-04-28

**Authors:** Shimaa H. A. Soliman, Aaron E. Stark, Miranda L. Gardner, Sean W. Harshman, Chelssie C. Breece, Foued Amari, Arturo Orlacchio, Min Chen, Anna Tessari, Jennifer A. Martin, Rosa Visone, Michael A. Freitas, Krista M. D. La Perle, Dario Palmieri, Vincenzo Coppola

**Affiliations:** 1 Department of Cancer Biology and Genetics, College of Medicine, The Ohio State University and Arthur G. James Comprehensive Cancer Center, Columbus, USA; 2 Department of Medicine, Dentistry and Biotechnology, G. d’Annunzio University of Chieti, Chieti, Italy; 3 Genetically Engineered Mouse Modeling Core, The Ohio State University and Arthur G. James Comprehensive Cancer Center, Columbus, USA; 4 Air Force Research Laboratory, Wright-Patterson AFB, 45433 Ohio, USA; 5 Department of Veterinary Biosciences and Comparative Pathology & Mouse Phenotyping Shared Resource, College of Veterinary Medicine, The Ohio State University and Arthur G. James Comprehensive Cancer Center, Columbus, 43210 Ohio, USA

**Keywords:** Mouse, Stress signalling

## Abstract

The lack of tools to reliably detect RanBP9 *in vivo* has significantly hampered progress in understanding the biological functions of this scaffold protein. We report here the generation of a novel mouse strain, RanBP9-TT, in which the endogenous protein is fused with a double (V5-HA) epitope tag at the C-terminus. We show that the double tag does not interfere with the essential functions of RanBP9. In contrast to RanBP9 constitutive knock-out animals, RanBP9-TT mice are viable, fertile and do not show any obvious phenotype. The V5-HA tag allows unequivocal detection of RanBP9 both by IHC and WB. Importantly, immunoprecipitation and mass spectrometry analyses reveal that the tagged protein pulls down known interactors of wild type RanBP9. Thanks to the increased detection power, we are also unveiling a previously unknown interaction with Nucleolin, a protein proposed as an ideal target for cancer treatment. In summary, we report the generation of a new mouse line in which RanBP9 expression and interactions can be reliably studied by the use of commercially available αtag antibodies. The use of this line will help to overcome some of the existing limitations in the study of RanBP9 and potentially unveil unknown functions of this protein *in vivo* such as those linked to Nucleolin.

## Introduction

RANBP9 is a scaffold protein whose biological functions are still unclear^1^. Since its initial description, it has been reported to interact with a number of proteins that are located in different cellular compartments and have different biological functions^1^. In many instances RANBP9 expression affects the half-life and stability of other proteins^1^. Most of these observations support the model in which RanBP9 is part of an E3 ligase multi-subunit structure called the CTLH (C-Terminal to LisH) complex that can induce substrate protein degradation via the proteasome. This E3 ligase structure is believed to be the equivalent of the GID (Glucose-Induced degradation Deficient) complex in *S. cerevisiae*, which is involved in responding to changes of nutrient availability in the microenvironment^2–5^. The human CTLH complex has been recently reported to be a heterodecameric structure that, in addition to RANBP9, includes its paralog RANBP10 and nine other poorly studied proteins (ARMC8, GID4, GID8, MAEA, MKLN1, RMND5A, RMND5B, WDR26, and YPEL5)^5–8^.

Objective difficulties impair the investigation of the cellular pathways and mechanisms in which RANBP9 takes part. Among other issues, the limited specificity and affinity of commercially available antibodies pose significant technical challenges. The existence of a paralog named RANBP10^9,10^ with extensive protein similarity to RANBP9 limits the RANBP9 specific sequence available to raise antibodies. In addition, some of the sequences might not have the ideal biochemical features for inducing a robust humoral immune response. Therefore, many of the commercially available αRANBP9 antibodies fall short in their detection power, reliability, and/or specificity.

We have previously used the only existing antibody validated by the Human Protein Atlas (HPA050007; www.proteinatlas.org) for immunohistochemical (IHC) detection of human RANBP9 in formalin-fixed paraffin embedded Non-Small Cell Lung Cancer (NSCLC) specimens^11^. Although specific for human RANBP9, in our hands HPA050007 does not seem to recognize mouse RanBP9 with similar affinity, especially in Western Blot (WB) in which signal-to-noise ratio is low. In addition, WB analysis often shows the presence of additional bands at lower molecular weight that might be due to non-specific binding.

We have used and will continue to use human cell lines for *in vitro* studies. However, to better recapitulate organismal physiology, a significant part of our ongoing investigation on RANBP9 involvement in tumor development and response to therapy necessarily takes advantage of *in vivo* murine models. In this regard, we had previously generated the constitutive RanBP9 knockout (KO) animal. On a hybrid C57Bl/6 x S129 genetic background, most homozygous KO mice were dying hours after birth. A small cohort of survivors showed small body size and severe sterility in both males and females^12^. These phenotypes were also confirmed by other groups^13,14^. Using reagents from the International Mouse Phenotyping Consortium (IKMC project nr: 44910; http://www.mousephenotype.org/), we have now engineered the conditional KO mouse that allows the study of RanBP9 loss of function *in vivo *and recapitulates the phenotype of the constitutive KO when the gene is ubiquitously deleted^15^. However, to overcome the limitations in the detection of endogenous RanBP9, we have decided to engineer a novel RanBP9 strain in which the wild type protein is fused to commonly used tags against which there are reliable and commercially available antibodies.

Here, we report the generation by CRISPR/Cas9 of the RanBP9-TT (= double Tag = TT) mouse line in which we have inserted a double epitope tag (V5-HA) at the C-terminus of the protein. We show that tagging of RanBP9 allows unequivocal detection of the protein both by IHC and biochemistry without affecting its essential biological functions. Thanks to the superior detection power intrinsic to the use of αtag antibodies, we found a previously unknown interaction of RanBP9 with the protein Nucleolin. Because we knocked-in the tags leaving intact the 3′-untranslated region (UTR) of the *RanBP9* genomic locus, the expression of the protein faithfully recapitulates the wild type (WT) expression. Therefore, the RanBP9-TT strain becomes a powerful tool to dissect *in vivo* the biology related to RanBP9 functions allowing its unequivocal detection in murine cells and tissues.

## Results

### Generation of the RanBP9-TT animals

**Figure 1:**
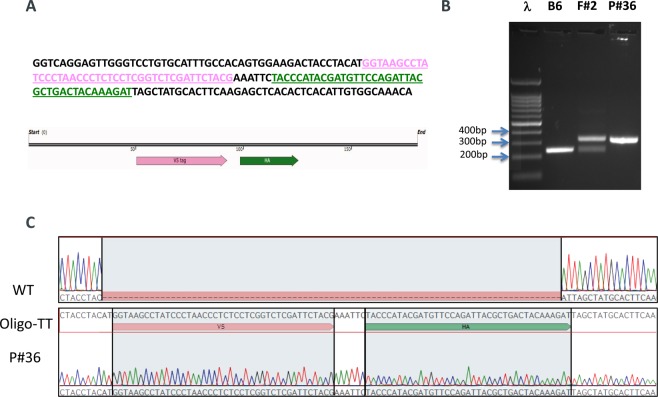
Generation of the *RanBP9-TT* mouse model by CRISPR/Cas9. (**A**) 180 bp single strand oligo DNA (ssODN) used as donor to recombine the V5 (PINK) and the HA (GREEN) tags into the C-terminus of RanBP9. (**B**) Representative PCR screening results from tail DNA of *WT* C57Bl/6 (negative control), *RanBP9-TT* Founder #2 (F#2), and homozygous *RanBP9-TT* pup number 36 (P#36). Results are congruent with prediction shown in Figure S1D. (**C**) Sanger-sequencing results from homozygous pup number 36 compared to C57Bl/6 WT and ssODN shown in A.

We used CRISPR/Cas9 to knock-in the double tag V5-HA at the C-terminus of RanBP9 (Fig. 1; Fig. S1). For targeting purposes, we employed the online Benchling software (https://www.benchling.com/). We selected the guide RNA (sgRNA) with the best specificity and efficiency scores closest to the insertion site before the stop codon (Fig. 1A and Fig. S1A–C). Pure C57Bl/6Tac WT fertilized eggs were used for the generation of founders (F0) mice. Two independent F0 animals (founder #1 and founder #2) were selected for further breeding and propagation of the RanBP9-TT colony. Both founders produced progeny (F1 mice) positive for the correct insertion of the double tag. Animals from both lines were phenotypically similar and were used for this work. Sanger sequencing showed that F1 animals from both founder lines contained the correct in-frame insertion of the V5-HA double tag (Fig. 1C). In order to significantly mitigate potential CRISPR/Cas9 off-targeting effects, we crossed F1 animals a second time with wild type C57Bl/6Tac mice to generate F2 progeny that were used for experimental purposes.

These results show that the V5-HA double tag at the C-terminus of endogenous RanBP9 was successfully inserted as designed.

### Addition of the V5-HA tag at the C-terminus does not cause lethality or infertility

On a mixed C57Bl/6 x S129 background using gene-trapped ES cells from the Baygenomics consortium^14,16^, homozygous inactivation of RanBP9 causes early postnatal lethality in mice^12^. The pure C57Bl/6 background seems to worsen the phenotype and homozygous KO newborns are rarely found, if any^14^.

**Figure 2:**
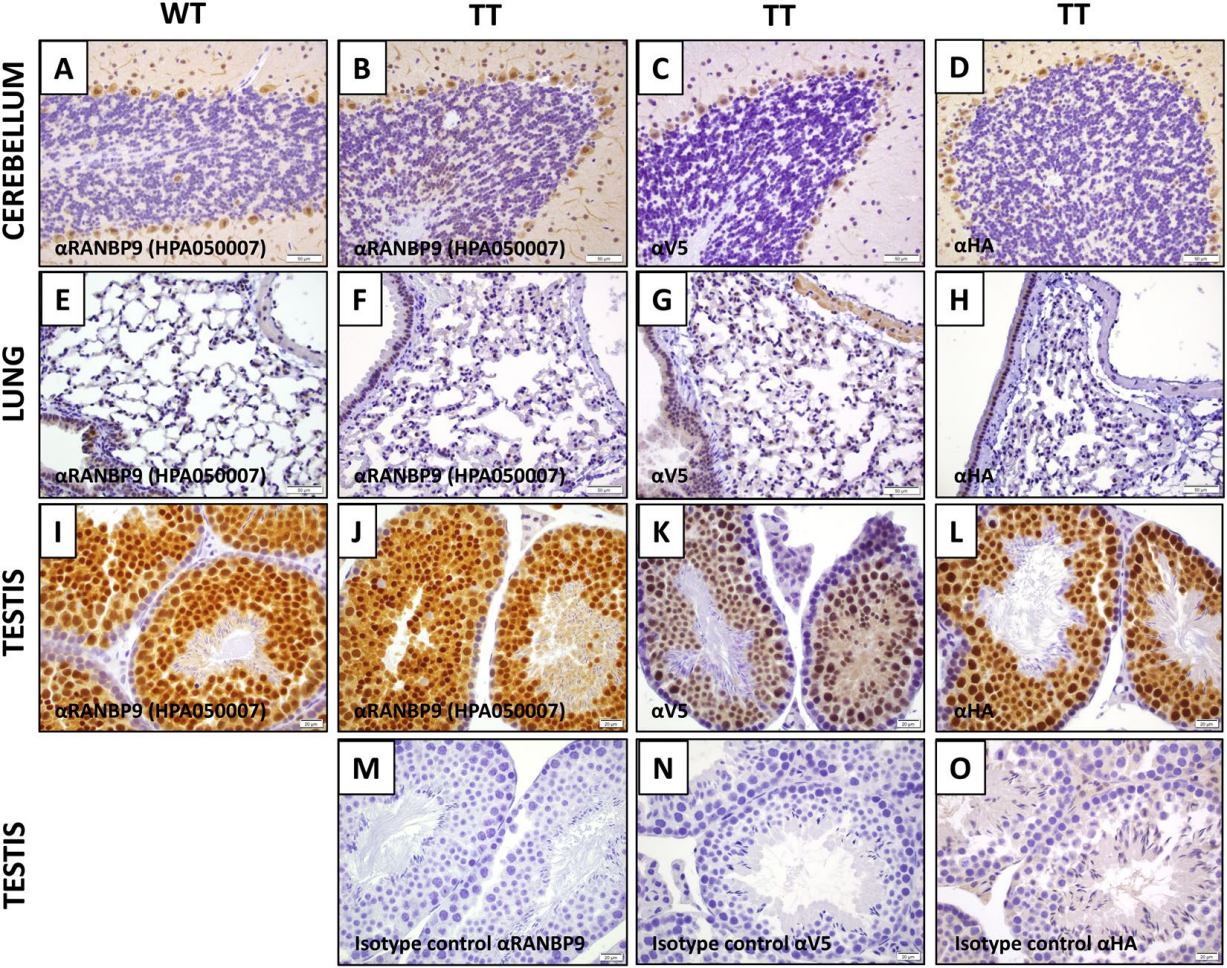
IHC detection of RanBP9 by αV5 in *RanBP9-TT* mice compared with detection by αRanBP9 specific antibody in *RanBP9-TT* and *WT* mice. (**A**–**D**) Cerebellum; (**E**-**H**); Lung; (**I**–**O**) Testis. Sections from indicated organs from *WT* mice (**A**,**E**,**I**) and *RanBP9-TT* mice (**B**, **C**, **D**, **F**, **G**, **H**, **J**, **K**, **L, M, N, O**) were stained with αRanBP9 HPA050007 antibody (**A**, **B**, **E**, **F**, **I**, **J**), or αV5 specific antibody (**C**,**G**,**K**) or αHA specific antibody (**D**,**H**,**L**). (**M**) Rabbit isotype control for αRanBP9. (**N**) Goat isotype control for αV5. (**O**) Rabbit isotype control for αHA. All pictures were taken with 40x objective and 10x eyepiece (400×). Scale bar = 50 um. EXCEPT: testis (60× = 600×, scale bar = 20 um).

We observed that *RanBP9-TT *homozygous knock-in (KI) mice are viable and do not show any obvious phenotype. They are born at Mendelian ratio and there are no differences in the number of females and males born. Adult animals do not show any gross anatomical or histological abnormality. Most importantly, homozygous RanBP9-TT males and females were bred to each other or to heterozygous and WT C57Bl/6Tac mice. Mice of both genders were able to reproduce in similar numbers to WT controls (Table 1). Testes and ovaries of *RanBP9-TT* mice show histological features similar to WT animals (Fig. 2 and Figs. S2,S3,S4). All together, these results show that the insertion of the V5-HA tag at the C-terminus of RanBP9 does not interfere with critical biological functions required for mouse development and survival. On the contrary, homozygous *RanBP9-TT* animals do not display any obvious phenotype and both male and female mice are fertile.

**Table 1:** *RanBP9-TT* mice are viable and fertile.

Female breeder genotype	x	Male breeder genotype	PROGENY GENOTYPE				
HET		HET		WT	HET	HOMO	Tot
			Females	2	5	5	12
			Males	7	5	1	13
			Tot	9	10	6	25
**HOMO**	**x**	**HET**		**WT**	**HET**	**HOMO**	**Tot**
			Females	0	8	1	9
			Males	0	2	7	9
			Tot	0	10	8	18
**HOMO**	**x**	**HOMO**		**WT**	**HET**	**HOMO**	**Tot**
			Females	0	0	4	4
			Males	0	0	6	6
			Tot	0	0	10	10

Pairs of parents of the indicated genotypes were set up for breeding. In all cases the expected Mendelian ratio of homozygous and heterozygous mutant mice was obtained. The number of male and female mice obtained from the different crosses was similar. Finally, both males and females homozygous RanBP9-TT mice were able to reproduce in numbers similar to WT C57Bl/6Tac present in the colony. A statistical analysis (Chi-squared test; GraphPad Prism 8) did not result in any significant difference between the expected and the observed number of animals of a specific genotype or gender.

### Addition of V5-HA double tag at the C-terminus allows faithful immunohistochemical detection of endogenous RanBP9

We then examined the expression of RanBP9 in mouse tissues by immunohistochemistry (IHC). We obtained tissue sections from *RanBP9 WT* and *RanBP9-TT* animals and stained them with αRanBP9 specific antibody (HPA050007), αV5, or αHA specific antibody. In Fig. 2, a selected array of tissues with significant expression of RanBP9 is shown. In *RanBP9-TT* Cerebellum, Testis, and Lung αV5 and αHA staining are similar in pattern but sharper than the αRanBP9 specific antibody. Immunoreactivity in the cerebellum is limited to the nucleus and cytoplasm of Purkinje cells. Intense nuclear and cytoplasmic staining is also apparent within the spermatocytes and round spermatids in seminiferous tubules of the testis. In the lung, labeling is detected in the cytoplasm of alveolar macrophages and the cardiac muscle normally present within pulmonary veins. On the other hand, αtag antibodies do not detect RanBP9 in sections of the same tissues from WT animals (Fig. S2). Finally, we also performed a more comprehensive IHC survey of several other organs/tissues using both αV5 (Fig. S3) and αHA (Fig. S4). Elsewhere in the nervous system, immunoreactivity is localized to the nucleus and cytoplasm of neurons in the cerebrum, CA1 and CA3 regions of the hippocampus, thalamus and brainstem, ganglia throughout the body, as well as the cytoplasm of choroid plexus epithelial cells. Cardiac and skeletal muscle is consistently immunoreactive, whereas smooth muscle labeling is variable and limited to the myometrium of the oviduct, uterus, small intestine and variably within arterioles. Squamous epithelial cells in the epidermis, hair follicles, forestomach and vagina display cytoplasmic staining. Epithelia throughout an array of other tissues also exhibit cytoplasmic staining including in: sebaceous glands (sebocytes), mammary glands (ducts), endometrium (including stroma), ovary (follicular and granulosa cells), oviduct, prostate gland, glandular stomach (parietal and mucous cells), small intestine, cecum (intensely immunoreactive), colon and the adrenal cortex. The nucleus of white adipocytes is positively stained in contrast to the diffuse cytoplasmic staining in brown adipocytes. Germinal centers within the spleen and lymph nodes exhibit nuclear staining in proliferating B lymphocytes. Hematopoietic cells such as megakaryocytes within the splenic red pulp also demonstrate cytoplasmic staining. Overt labeling was not detected in the kidney, urinary bladder, liver or pancreas.

### V5-HA-tagged RanBP9 mRNA and protein expression is similar to wild type RanBP9

**Figure 3:**
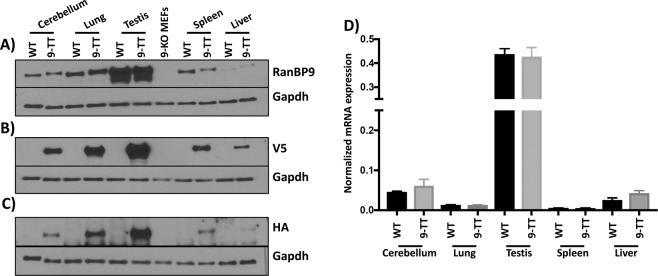
Protein and transcript expression of RanBP9 in selected adult *RanBP9 WT* and *RanBP9-TT* (9-TT) mouse tissues. Proteins were extracted from mouse cerebellum, lung, testis, spleen, and liver of *RanBP9 WT* and *RanBP9-TT* mice. In addition, proteins were also extracted from *RanBP9-KO* MEFs (9-KO MEFs) and used as negative control. (**A**) Detection of RanBP9 wild type and V5-HA-tagged by αRanBP9 specific antibody. (**B**) Detection of RanBP9 wild type and V5-HA-tagged by αV5 specific antibody. (**C**) Detection of RanBP9 wild type and V5-HA-tagged by αHA specific antibody. (**D**) Measurement of RanBP9 mRNA by RT-PCR. Expression levels of RanBP9 were assessed in Cerebellum, Lung, Testis, Spleen, and Liver from *RanBP9 WT* mice and *RanBP9-TT* (9-TT) mice. Expression levels in WT and 9-TT tissues are represented in black and grey bars respectively. Each sample was analyzed in triplicate. Each well was normalized to average values of β-actin to obtain a ΔCt = 2^-(FAM dye Ct-averageActb dye Ct)^. mRNA levels in each WT tissue were similar to their counterpart 9-TT tissues. A statistical analysis was performed using GraphPad Prism 8 to compare expression values in same organs from WT and 9-TT mice. The Mann-Whitney (Wilcoxon rank sum) test was used and none of the values between WT and 9-TT levels were significantly different.

Next, we sought to measure the amount of RanBP9-TT protein and messenger RNA in different tissues by WB and qRT-PCR, respectively. We obtained protein cell extracts from tissues and organs with various levels of reported expression^12^. WB analysis probing with antibodies specific to RanBP9 (rabbit Abcam ab140627) (Fig. 3A), or αV5 (Fig. 3B), or αHA (Fig. 3C) shows that the levels of expression of RanBP9-TT are similar to the levels of RanBP9 in wild type animals. Accordingly, we did not detect any significant difference between levels of RanBP9 mRNA in wild type and *RanBP9-TT* mice (Fig. 3D).

These results show that the expression of RanBP9-TT is similar to wild type RanBP9 and there are no perturbations of expression caused by the insertion of the V5-HA tag.

### RanBP9-TT protein maintains the known RanBP9 WT interactions

**Figure 4:**
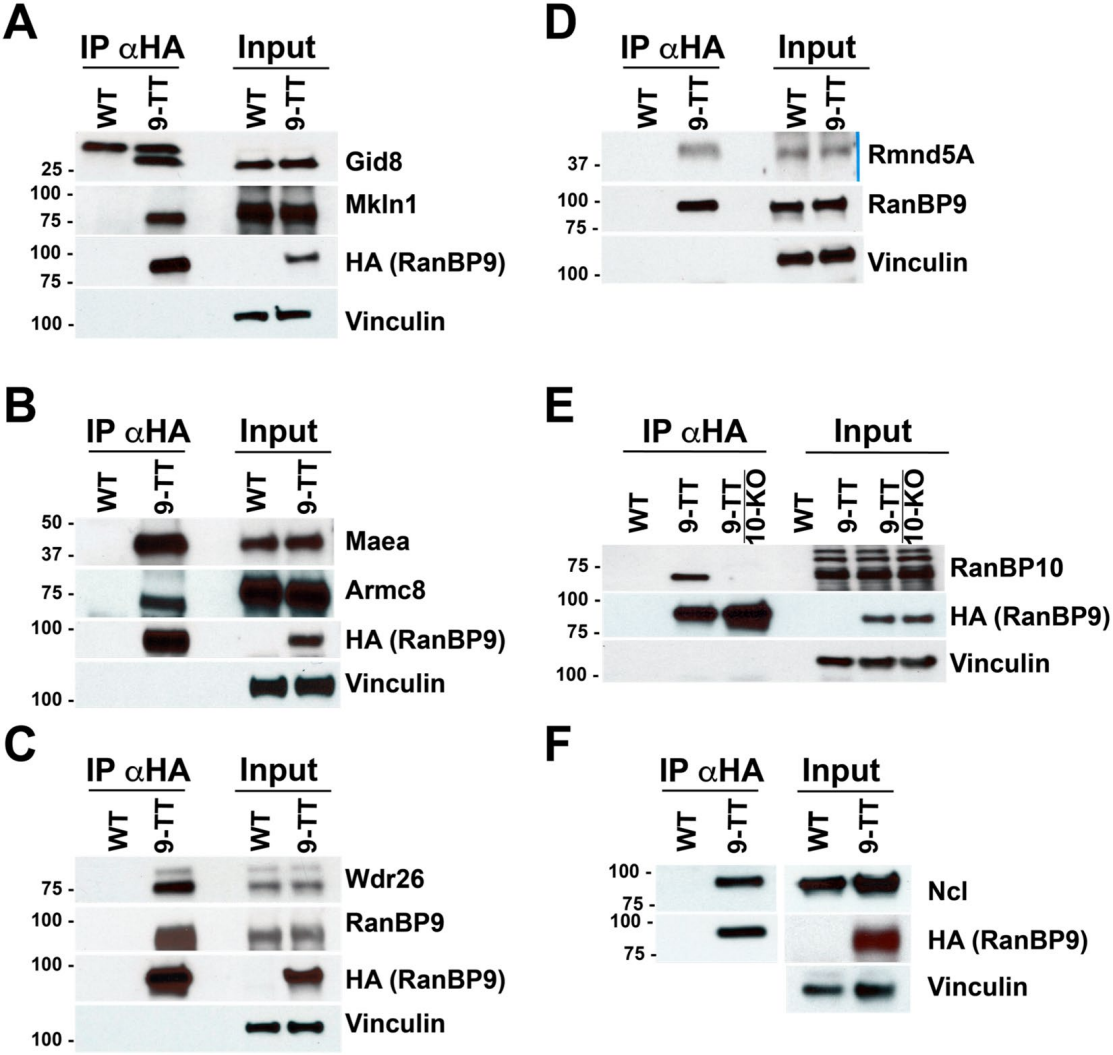
V5-HA-tagged RanBP9 maintains its ability to interact with known members of the CTLH complex and Nucleolin. *RanBP9 WT* and *TT* immortalized Mouse Embryonic Fibroblasts (MEFs) were cultured in standard conditions and protein lysates were obtained. Resin conjugated with αHA antibodies was used to immunoprecipitate RanBP9-TT protein. IPed fractions and 5% of input were run on gels to generate 5 different membranes that were probed with the indicated antibodies by WB. Vinculin is used as loading control. Shown results are representative of two independent experiments (biological replicates).

RanBP9 is part of a poorly studied multi-subunit complex called the CTLH complex^6^. Therefore, we sought to determine whether the addition of the double V5-HA tag at the C-terminus of RanBP9 interfered with its binding to the CTLH complex. To this aim, we generated homozygous *RanBP9-TT* mouse embryonic fibroblasts (MEFs) and performed immunoprecipitation (IP) using resin-conjugated αHA antibodies. We ran four different gels and we probed the immunoprecipitated (IPed) fractions by WB. We were able to purchase specific antibodies for seven members of the CTLH complex. Gid8 and Muskelin are clearly present in IPed fractions from *RanBP9-TT *but not from *WT* MEFs (Fig. 4A). Similarly, Maea, Armc8 (Fig. 4B), Wdr26 (Fig. 4C) and Rmnd5A (Fig. 4D) are present only in fractions pulled down by resin-conjugated αHA in the *RanBP9-TT *MEFs and not *WT* MEFs. The paralog of RanBP9, RanBP10, is clearly present only in *RanBP9-TT* MEFs and not in *WT* MEFs immunoprecipitated fractions with resin-conjugated αHA and appears as a sharp band of the expected molecular weight (Fig. 4E). However, due to the poor sensitivity and specificity of commercially available αRanBP10 antibodies, to prove that it is RanBP10 that is immunoprecipitated and not a non-specific protein of similar size, we also extracted and immunoprecipitated lysates from *RanBP10* knock-out/*RanBP9-TT *double mutant MEFs. Data unequivocally show the disappearance of the protein immunoprecipitated from *RanBP10 WT*/*RanBP9-TT* MEF cell lysates and indirectly demonstrate its specificity (Fig. 4E).

Taken together, these results clearly indicate that V5-HA-tagged RanBP9 interacts with known partners of WT RanBP9. However, since we were not able to probe by WB for the remaining members of the CTLH complex, we decided to perform a proof-of-principle small-scale IP using αV5 antibody followed by high-resolution mass spectrometry (MS) analysis (IP-MS/MS, for complete raw data, see supplementary material). Initially, we decided to perform this analysis on nuclear- and cytoplasmic-enriched fractions to better pinpoint interactions. However, the amount of nuclear extracts was low and only yielded a very limited number of peptides. Therefore, we concentrated our attention on cytoplasm-enriched fractions only. Also, ourIP-MS/MS experiments included two different negative controls: lysates from untreated *RanBP9 WT* MEFs and lysates from cross-linked *RanBP9 WT* MEFs. In all instances, the amount of protein extract was limited. In our data elaboration, we decided to use the most stringent criteria for selection of putative interactors of RanBP9 and ranked all identified proteins by abundance in cytoplasmic-enriched fractions of *RanBP9-TT* MEFs but absent in both untreated and cross-linked *RanBP9 WT* ones (Table 2). The only exception to this rule was Muskelin, which presented one count in cross-linked *RanBP9 WT *immunoprecipitate. Table 2 shows that RanBP9 is robustly enriched and ranks first for abundance in *RanBP9-TT *fractions while is absent in the*RanBP9 WT* MEF IPed extracts. Importantly, the list of proteins detected in *RanBP9-TT* lysates by IP-MS/MS includes all of the remaining 10 members of the CTLH complex within the top 17 hits.

**Table 2:** List of top hits of IP-MS/MS from RanBP9-TT MEFs following ranking by abundance.

RANK	UNIPROT ID	PROTEIN NAME	Note (Ref)	Protein abundance	Protein abundance	Protein abundance
				BP9TT	WT	WT cross-linked
1	P69566	RanB9	CTLH	25	0	0
2	O89050	Mkln1	CTLH	17	0	1
3	Q8C6G8	Wdr26	CTLH	10	0	0
4	Q9D7M1	Gid8	CTLH	10	0	0
5	Q9DBR3	Armc8	CTLH	10	0	0
6	P22777	Pai1		8	0	0
7	Q4VC33	Maea	CTLH	8	0	0
8	Q80YX1	Tena		7	0	0
9	Q6VN19	RBP10	CTLH	4	0	0
10	Q80YQ8	Rmd5A	CTLH	4	0	0
11	P21981	Tgm2		4	0	0
12	Q9CPY6	Gid4	CTLH	4	0	0
13	P62700	Ypel5	CTLH	3	0	0
14	Q61001	Lama5		2	0	0
15	Q99K41	Emil1		2	0	0
16	Q9R118	Htra1		2	0	0
17	Q91YQ7	Rmd5B	CTLH	2	0	0
18	P04104	K2C1		1	0	0
19	P62274	RS29		1	0	0
20	O08638	Myh11		1	0	0
21	P01786	Hvm17		1	0	0
22	Q6PGN1	ERFE		1	0	0
23	Q8CIH5	Plcg2		1	0	0
24	Q64339	Isg15		1	0	0
25	Q9JIY5	Htra2	(*)	1	0	0
26	O08807	Prdx4		1	0	0

With the exception of Mkln1 (known CTLH member), only protein hits that did not show any counts both in the non-cross-linked or cross-linked *RanBP9 WT* MEFs are listed. Protein ID, name, and number of PSMs of immunoprecipitated proteins from *RanBP9-TT* and *WT* MEFs are reported. CTLH = known member of the CTLH complex. (*) Mouse homolog of HTRA2 protein found in the 2018 study by Lampert *et al*. as potential interactor of both RMND5A and ARMC8.

Taken all together, these results indicate that the addition of the V5-HA to RanBP9 C-terminus does not preclude its participation in the CTLH complex.

### RanBP9-TT protein interacts with Nucleolin

Previous studies have found potential interactions of RANBP9 using commercially available antibodies for IP-MS/MS approaches^5,6^. Most of these studies were performed in human cells and using different antitag antibodies. One study was performed in testes and revealed the interaction of RANBP9 with several RNA processing factors^13^. In examining the list of proteins from our IP-MS/MS experiment in the cross-linked samples, we noticed that one potential unknown interactor of RanBP9 and object of investigation in our laboratory could be Nucleolin (Ncl) (for complete raw data, see supplementary material). Therefore, we probed fractions immunoprecipitated by αHA-conjugated resin by WB using an anti-Ncl specific antibody. In accordance with the proteomic results, we were able to detect the presence of Ncl in lysates from *RanBP9-TT* cells but not from *RanBP9 WT* MEFs (Fig. 4F). These results reveal a novel interaction between RanBP9 and Ncl.

## Discussion

Specific and reliable detection of proteins *in vivo* is difficult and it is estimated that researchers waste millions of dollars every year on the use of non-specific antibodies^17,18^. The obstacles are insurmountable when the proteins of interest are not commonly studied and thus validated reagents are limited or missing. In some instances, the existence of paralogs with extensive homology further complicates the generation of tools that can unequivocally detect only the specific target. This is the case for the poorly studied scaffold protein RanBP9 and its paralog RanBP10, which share high homology in 4 out of the 5 annotated protein-protein interaction domains^9,10^. To date, there is only one validated antibody for detectionby IHC of human RANBP9 as reported in the Human Protein Atlas project (www.proteinatlas.org). This reagent (HPA050007) is not validated for detection of mouse RanBP9. In our hands, this antibody did not appear to have the sensitivity at levels similar to antibodies from other sources that performed better for biochemical detection of both human and mouse RanBP9 by conventional methods such as IP and WB. While it maybe necessary to use multiple antibodies for the detection of a target protein, this necessity generates frustration due to the quality of results and cost of reagents.

To overcome these limitations and have in hand a reliable tool for the study of RanBP9 in physiological and pathological paradigms *in vivo*, we decided to use CRISPR/Cas9 and generate *RanBP9-TT* mice by the addition of a double V5-HA tag at the C-terminus of RanBP9. Biochemical tags are increasingly an excellent solution for detection problems and are widely employed for research purposes^19^.

In order to minimize steric hindrance, we chose to use relatively small tags of 9 amino acids each. In addition, we also chose to use two of them (V5 and HA) to increase the detection power and provide two different options.

The engineering of *RANBP9-TT* mice was possible thanks to CRISPR/Cas9. In fact, compared to the classical use of ES cells^20^, in addition to being faster and efficient in various genetic backgrounds, this new technology offers the critical advantage of not leaving any “scar” in the edited allele other than the desired engineered mutation^21,22^. Therefore, in *RanBP9-TT* mice, the C-terminus of RanBP9 as well as the 3′-UTR remained intact, preserving not only the protein structure, but also the endogenous regulation of expression (Fig. 1 and Fig. S1). The WB analysis in Fig. 3A-C clearly shows that RanBP9 protein levels in all analyzed organs/tissues are comparable between *RanBP9 WT* and *TT* mice. Interestingly, detection of RanBP9 by the use of αV5 (Fig. 3B) appears to have superior sensitivity and specificity (lower background) compared not only to the αHA (Fig. 3C), but also the αRanBP9 specific antibody (Fig. 3A). In particular, it is worth to point out the augmented sensitivity in liver samples of the αV5/αHA-tag based detection that yielded a very clear signal in comparison to the one using RanPB9-specific antibody, which showed barely detectable bands (Fig. 3A *vs*. Figure 3B). qRT-PCR measurements confirmed that there are no differences in *RanBP9* mRNA levels in different organs/tissues from *RanBP9-TT* compared to *RanBP9 WT* mice (Fig. 3D). However, it is interesting to note that levels of mRNA do not always correlate with protein levels. While testis yielded the highest amount of protein and mRNA, lung exhibited the second highest level of protein but displayed a level of messenger lower than cerebellum, where RanBP9 protein was significantly less abundant. Also, liver showed the lowest amount of RanBP9 protein, but a level of mRNA higher than both spleen and lung. These results might be explained as technical artifacts due to differences in sample lysing and extraction or, alternatively, suggest the existence of post-transcriptional regulatory mechanisms of gene expression. In this regard, we have previously observed this poor correlation between levels of *RanBP9* mRNA and protein in human NSCLC samples^11^. This phenomenon leads us to believe that it is always necessary in cancer cells and tissues to measure protein levels to evaluate the expression of *RanBP9* rather than relying on the amount of RNA. Therefore, a tool like the *RanBP9-TT* mice, which permits the unequivocal detection of RanBP9 protein, becomes invaluable for *in vivo* studies. Indeed, the IHC analysis shown in Fig. 2, Figs. S2,S3 and S4 provided strong evidence that the *RanBP9-TT* strain can be used to clearly identify cells expressing this protein in all tissues. When crossed to *in vivo* models of different malignancies, *RanBP9-TT *mice will be of value in studying the role of this protein during tumorigenesis. One priority for our group is to show the relevance of this protein is in lung tumorigenesis and its specific expression in tumor cells versus cell populations in the microenvironment. Further, we have shown that DNA damage elicits RANBP9 post-translational modifications and induces change of its relative subcellular localization^23^. Given that studies *in vitro *completely miss the complexity of organismal responses and adaptive mechanisms that operate *in vivo*, we plan to use our new mouse model to study how the protein is modified and whether its localization changes following genotoxic stress in the whole body, for example.

We have deliberately decided the placement of the TT tag based on *in vitro* evidence from our lab and other groups indicating that C-terminus tagging of RanBP9 was not impairing RanBP9 movement inside cells upon specific stimulation^23–25^. However, such a strategy still presents the intrinsic and real risk that the added tag might block RanBP9 interactions with other proteins thus interfering with its biological functions. Here, we show that vital functions and known interactions of RanBP9 are preserved in *RanBP9-TT* mice. In fact, *RanBP9* constitutive KO mice show a severe perinatal lethality and the occasional survivors, both males and females, are runts and sterile with gonads completely devoid of germ cells^12,14^. On the other hand, *RanBP9-TT *homozygous animals did not show any obvious phenotype. A basic observational and anatomical examination showed that *RanBP9-TT* knock-in animals develop and grow similarly to WT littermates. Gross anatomic parameters and reproductive performance were similar in *RanBP9-TT* mice compared to WT littermates (Table 1). Specifically, *RanBP9-TT* mice displayed normal histological architecture of both testis and ovary, among other tissues. Sperm and oocytes were present in *RanBP9-TT* animals without any appreciable difference compared to WT controls (Fig. 2, Figs. S2,S3,S4). Since in germ cells RanBP9 is expressed at its highest levels, these results clearly show that the TT tag does not have undesired deleterious effects, nor does it interfere with fundamental biological functions of RanBP9 necessary for sperm and oocyte development and maturation^12,26^.

Obviously, it is impossible for us to completely exclude the possibility that the V5-HA tag is interfering at the molecular level with yet unknown biological functions of RanBP9. However, this protein is part of an evolutionary conserved and ubiquitously expressed multi-subunit E3-ligase called the CTLH (C-Terminal to LisH) complex^4,6–8^. Therefore, a valid, if not the best, strategy for us to test whether the TT tag interfered with molecular interactions entertained by RanBP9 was to show whether the V5-HA-tagged RanBP9 protein was incorporated into the CTLH complex. Here, we show by WB analysis that the immunoprecipitation of tagged RanBP9 by αHA brings down Gid8, Muskelin (Fig. 4A), Maea, Armc8 (Fig. 4B), Wdr26 (Fig. 4C), Rmnd5A (Fig. 4D), and RanBP10 (Fig. 4E). Identification of 7 of the 10 known CTLH members (not including RanBP9) strongly indicated that the tagged protein is incorporated into the complex and retains its activity and functionality. As reliable antibodies for the remaining CTLH members (Gid4, Rmnd5B, and Ypel5) are not commercially available, to further validate our findings, we performed IPs using αV5 from *RanBP9-TT* and *WT* MEF cell extracts followed by tandem mass spectrometry (Table 2). Ranking proteins by number of peptide spectral matches (PSMs) or counts in the *RanBP9-TT* samples and excluding those with counts in the IP from *RanBP9 WT* cells (both non-cross-linked and cross-linked), all 11 members of the CTLH complex are present within the top 20 hits (Table 2). This proof-of-principle experiment, performed in duplicate with and without cross-linking, demonstrated that the entire complex was immunoprecipitated by V5-HA-tagged RanBP9. Hence, IP-MS/MS data corroborated the WB results (Fig. 4A-C) and added further evidence that RanBP9-TT conserves the same interactions of the endogenous RanBP9 WT protein.

However, it needs to be clear that the proteomic study presented here aimed only to show the expected interactions of RANBP9-TT protein with the CTLH complex members. Therefore, it was limited in scope and carried out on a small amount of protein extracts. Although performed pulling down RanBP9 in immortalized mouse fibroblasts, it is interesting to see the presence of Htra2 (Htra Serine Protease 2; hit number 25 of the list in Table 2). In fact, this protein is one of the top proteins identified as potential interactor of both RMND5A and ARMC8 by Lampert*et al*.^6^ in their study of the human CTLH complex. This finding suggests that the CTLH-HTRA2 interaction is conserved in mouse and human and its biological significance should be investigated.

The IP-MS/MS list, included several unknown potential interactors of RanBP9. Due to our limited nature of our proteomic investigation, all of the listed novel potential interactors will need to be confirmed by more in-depth experiments. Here, we focused only on confirming by IP-WB the interaction between RanBP9 and Ncl revealed in the cytoplasm-enriched RanBP9-TT (but not WT) immortalized MEF fractions after cross-linking (Fig. 4F). This nucleolar protein is of particular interest to us and other groups for several reasons. First, due to its aberrant cytoplasmic and cell-membrane localization specifically in cancer cells, Ncl represents an ideal target for cancer therapy in several aggressive types of malignancies^27,28^. Second, Ncl is an RNA-binding protein involved in processing and stabilization of coding and non-coding RNA, with this interaction RanBP9 could have in somatic cells a potential involvement of in RNA metabolism, which has been already hypothesized in germ cells^13^. Third, Ncl is involved in the response to DNA damage and might represent another unknown link between RANBP9 and p53 in cells treated with genotoxic agents. In fact, we have previously shown that when RANBP9 is ablated in NSCLC cells the levels of p53 are robustly decreased in cells treated with CDDP or IR^11,23^. On the other hand, Ncl together with the ribosomal protein L26 bound to the 5′ untranslated region of the p53 mRNA increases the translation and consequently the levels of p53 protein^29^. Therefore, it is tempting to speculate that in response to damage of the DNA, Ncl and RanBP9 cooperate in elevating and maintaining adequate levels of p53 stabilizing its transcript. However, the mechanisms of Ncl-RanBP9 interaction will need to be confirmed in and studied in more depth. Therefore, those experiments are beyond the scope of the present investigation, which aimed only to validate our novel *RanBP9-TT *strain.

In summary, we show here that we have engineered a new mouse model bearing a tagged RanBP9 protein that retains all the known features and abilities of the endogenous wild type molecule. The TT tag allows reliable immunohistochemical detection and enrichment of RanBP9 together with its known binding partners of the CTLH multi-subunit complex. This new tool will be invaluable for the study of RanBP9 at the organismal level in a variety of in physiological and pathological conditions and possibly reveal unknown interactions and molecular functions.

## Material and Methods

### CRISPR/Cas9 RanBP9-TT mouse generation and analysis

Mouse experiments were performed according to The Ohio State University Institutional Animal Care and Use Committee (IACUC) guidelines after a review of an institutional review board. Work performed for the present study is described in IACUC approved protocol number 2008 A0009-R3 titled “Generation, analysis and training in the use of gene knock-out and transgenic rodents”; Principal Investigator: V. Coppola.

The Genetically Engineered Mouse Modeling Core of the Ohio State University Comprehensive Cancer Center generated *RanBP9-TT* mice by using CRISPR/Cas9 technology. Briefly, CRISPR targeting strategy was designed using the online algorithm at www.benchling.org. The synthetic single strand oligo donor DNA (ssODN) in which both V5-HA tags are contained was purchased from Integrated DNA Technologies (Coralville, Iowa, USA). Synthetic tracrRNA and crRNA was purchased from Sigma-Aldrich (Saint Louis, MO, USA). GeneArt Platinum Cas9 nuclease purified protein (cat. nr. B25642) was purchased from Invitrogen (ThermoFisher Scientific; Waltham, MA, USA). The mix of assembled sgRNA with Cas9 protein (RNP complex) and ssODN was microinjected into C57Bl/6Tac zygotes. C57Bl/6Tac WT animals were used for founder mating purposes.

### Immunohistochemical (IHC) analysis of RanBP9-TT and RanBP9 WT expression in mouse adult tissues

Adult mouse tissues were sectioned and stained by the Comparative Pathology &Mouse Phenotyping Shared Resource of the Ohio State University Comprehensive Cancer Center as previously described^30^. Briefly, tissues were fixed in 10% (v/v) formalin, routinely processed, and embedded in paraffin for immunohistochemical characterization.

Formalin-fixed, paraffin-embedded tissues were cut into 4-μm-thick sections, were dewaxed in xyleneand rehydrated through graded ethanol solutions to water. Antigens were retrieved by heating the tissue sections at 96–98 °C for 25 minutes in citrate solution (10 mmol/L, pH 6.0). Sections were cooled and immersed in methanol in the presence of 0.3% hydrogen peroxide for 15 minutes to block the endogenous peroxidase activity. After being rinsed first in tap water and then in PBS for 5 minutes, sections were subsequently incubated with the indicated antibodies (rabbit αRanBP9 antibody, 1:50, Sigma-Aldrich cat. nr. HPA050007; goat αV5 antibody, 1:350, Abcam cat. nr. ab95038; and, rabbit αHA, 1:800, Cell Signaling cat. nr. C29F4) at 4 °C overnight. As negative controls, slides were incubated with rabbit or goat IgG instead of the primary antibody. The sections were rinsed with buffer and then incubated with horseradish peroxidase-labeled secondary antibody (RanBP9: αrabbit, 1:1000; V5: αgoat, 1:500; HA: αrabbit, 1:500) for 30 minutes. 3, 3′-Diaminobenzidine (DAB) was used as the chromogen, and hematoxylin as the nuclear counterstain. Sections were then dehydrated, cleared and mounted permanently with glass coverslips. Slides were photographed with an Olympus BX45 light microscope with attached DP27 digital camera and corresponding CellSen software (B&B Microscopes Limited, Pittsburgh, PA).

### WB analysis of RANBP9 expression

Mouse tissues were homogenized on ice in NP-40 buffer supplemented with Halt Protease and Phosphatase Inhibitor cocktail (Thermo Fisher Scientific cat. nr. 78442). Protein concentration was determined using the Bio-Rad protein assay dye (Bio-Rad cat. nr. 5000006). Western blot analysis was performed using 30 to 50 μg proteins run on Mini-PROTEAN TGX precast gels (Bio-Rad cat. nr. 456–1094).

αRANBP9 primary antibody was purchased from Abcam (cat. nr. ab140627) and used at a dilution of 1:2,000 in 5% non-fat milk in TBS-T. Signals were detected with HRP-conjugated secondary antibodies (GE Healthcare) and the chemiluminescence substrate Supersignal wet pico PLUS (Thermo Fisher Scientific). Equivalent loading among samples was confirmed with αGAPDH (Cell Signaling cat. nr. 3683S).

### RT-PCR analysis of RanBP9 expression

Murine tissues were homogenized on ice using 1 ml TRIzol Reagent (Life technologies cat. nr. 15596018) following the manufacturer’s protocol. The extracted RNA was quantified using Nano-Drop 2000. To analyze *RanBP9* gene expression, real time quantitative polymerase chain reaction (RT-qPCR) was performed from complementary DNA (cDNA). Total RNA (500 ng) was treated with DNAse I (Invitrogen cat. nr. 18068015) and used for reverse transcription with the High Capacity cDNA Reverse Transcription Kit (Applied Biosystem cat. nr. 4368814) following the manufacturer’s instructions. 20 ng of cDNA per reaction were used for RT-qPCR with TaqMan fast advanced master mix (Applied Biosystems cat. nr. 4444557) following the manufacturer’s instructions. Samples were amplified simultaneously in triplicate in one assay run using the StepOnePlus^TM^ real time PCR system. The RanBP9 probe used was Mm00451306_m1. Beta Actin was used as reference gene (probe Mm00607939_s1). Analysis was performed by Prism 8 software (GraphPad Prism®) using the Δ-ct method (Applied Biosystems).

### Immunoprecipitation

RanBP9 was immunoprecipitated from total protein extracts using monoclonal αHA-Agarose antibody (Sigma cat. nr. A2095-1ML). Briefly, 1 mg of total protein extracts were pre-cleared by incubating with Pierce Protein A/G Plus Agarose (Thermo Scientific cat. nr. 20423) for 1 hr at 4 °C. Lysates were centrifuged at 4 °C; the supernatant was collected and incubated with 5 μg of the primary antibody overnight at 4 °C. Proteins interacting with RanBP9 were detected in the immunoprecipitates of RanBP9 using wet-transfer WB. Interactors of the CTLH complex were detected using the following antibodies: αRMND5A (Novus Biologicals cat. nr. NBP1-92337), αWDR26 (Abcam cat. nr. ab85962), αARMC8 (Proteintech cat. nr. 12653-1-AP), αMuskelin (Proteintech cat. nr. 14735-1-AP), αC20orf11 (a.k.a. GID8; Proteintech cat. nr. 24479-1-AP), αMAEA (R&D Systems cat. nr. AF7288), αRanBP10 (Millipore SIGMA cat. nr. SAB3500163), and αNucleolin (D4C7O; Cell Signaling rabbit mAB cat. nr. 14574).

### Immunoprecipitation tandem mass spectrometry (IP-MS/MS)

Cells were separated into cytoplasmic and nuclear fractions prior to immunoprecipitation as described in Mohammed *et al*.^31^ with ad hoc modifications. Briefly, *RanBP9 WT* and *RanBP9-TT* MEFs (15×10^6^) were washed and harvested with PBS either left untreated or after crosslinking with 1% formaldehyde (Thermo Fisher Scientific cat.nr. 28908) for 8 min and quenched with glycine (0.1 M). Pelleted fractions were resuspended in lysis buffer 3 (LB3–50 mM Tris-HCL, pH 7.5, 250 mM NaCl, 1 mM EDTA, 0.5 mM EGTA, 0.1% (w/v) sodium deoxycholate, 0.5% (v/v) N-laurylsarcosine) supplemented with inhibitors and sonicated with a focused ultrasonicator (AFA Covaris E220 Evolution). Following centrifugation, 1 mg of cytoplasmic or nuclear protein extract was incubated with 25 μL of Pierce Protein A/G Magnetic Beads (Thermo Fisher Scientific cat. nr. 88803) pre-bound to αV5 antibody (5 μg, Invitrogen cat. nr. R96025) at 4 °C with rotation overnight. After stringent washes with RIPA buffer to remove non-specific proteins, beads were equilibrated in 100 mM ammonium bicarbonate with 3 washes prior to on-bead digestion with trypsin (800 ng; Promega cat. nr. V5280) overnight at 37 °C, 800 rpm. Digestion continued the next day for 4 hours after samples were supplemented with an additional bolus of trypsin (800 ng). Beads were placed in a magnetic stand to collect supernatants and dried down in a Speedvac concentrator. Prior to mass spectrometry analysis, peptides were resuspended in loading buffer (2% ACN, 0.003% TFA) and quantified via Nanodrop. Chromatography separation and mass spectrometry methods were performed as described in Scheltema *et al*. for data dependent acquisition on a Q-Exactive HF (Thermo Fisher Scientific)^32^. Tryptic peptides (1000 ng) were isocratically loaded onto a PepMap C18 trap column (300 µm x 5 mm, 100 Å, 5 µm) at 5 µL min^−1^ and analytical reversed phase separations were performed on a DionexUltiMate 3000 RSLCnano HPLC system coupled to an EASYSprayPepMap C18 column (15 cm × 50 µm ID, 100 Å, 2 µm) over a 90 min gradient at 300 nL min^−1^. RAW mass spectrometry files were converted to mzml and searched against a complete, reviewed mouse Uniprot database containing common contaminants (downloaded 04/10/2019) via OpenMS (version 2.3.0) with X! TANDEM (release 2015.12.15.2) and MS-GF + (release v2018.01.20).

### Statistical analysis

Statistical analysis was performed using GraphPad Prism 8 (GraphPad, San Diego, CA). We performed a contingency table analysis of the data reported in Table 1 showing the number of male and female live mice from different crossings with three different genotypes (*WT*, *RanBP9TT HET*, *RanBP9TT HOMO*) by using a Chi-square (and Fisher’s exact) test with a confidence interval of 95%. The analysis did not result in any significant difference between the expected and the observed number of animals of a specific genotype or gender. To compare expression values in same organs from WT and TT mice showed in Fig. 3, we performed a Mann-Whitney (Wilcoxon rank sum) non-parametric test without assuming a normal distribution of values. Results of the analysis indicated that none of the values between WT and TT levels were significantly different.

### RanBP9-TT mouse availability

*RanBP9-TT* knock-in mice are available from the corresponding author on reasonable request and contingent to a Material Transfer Agreement with The Ohio State University.

## Supplementary information

Supplementary Information
