# Supplementary material for: Tagging enhances histochemical and biochemical detection of Ran Binding Protein 9 in vivo and reveals its interaction with Nucleolin
Source: Sci Rep. 2020 Apr 28;10:7138. doi: 10.1038/s41598-020-64047-8 (PMC7188826; doi:10.1038/s41598-020-64047-8)
Supplement: Supplementary file 1 — Supplementary Information [file 41598_2020_64047_MOESM1_ESM.pdf]

Supplementary Material

Tagging enhances histochemical and biochemical detection of Ran Binding Protein 9 *in vivo* and reveals its interaction with Nucleolin

Shimaa Soliman[1, 2#], Aaron E. Stark[3, #], Miranda L. Gardner[1], Sean W. Harshman[4], Chelssie C. Breece[5], Foued Amari[3], Arturo Orlacchio[1], Min Chen[3], Anna Tessari[1], Jennifer A. Martin[4], Rosa Visone[2], Michael A. Freitas[1], Krista M.D. La Perle[5], Dario Palmieri[1], and Vincenzo Coppola[1, 3, *].

[1]Department of Cancer Biology and Genetics, College of Medicine, The Ohio State University and Arthur G. James Comprehensive Cancer Center; [2]Department of Medicine, Dentistry and Biotechnology, G. d'Annunzio University of Chieti, Italy; [3]Genetically Engineered Mouse Modeling Core, The Ohio State University and Arthur G. James Comprehensive Cancer Center; [4]Air Force Research Laboratory, Wright-Patterson AFB, 45433, Ohio, USA; [5]Department of Veterinary Biosciences and Comparative Pathology & Mouse Phenotyping Shared Resource, College of Veterinary Medicine, The Ohio State University and Arthur G. James Comprehensive Cancer Center, Columbus, 43210, Ohio, USA.

Supplementary Figures

Figure S1. Generation of the *RanBP9-TT* mouse model by CRISPR/Cas9. **A**) Depiction of guide RNA (sgRNA; light GREEN) selected to target the last codon of *RanBP9* and relative on-target (MAROON) and off-target (LIGHT BROWN) scores (Benchling). **B**) sgRNA sequence and scores (Benchling). **C**) Predicted off-target sites and scores using the selected sgRNA (Benchling). **D**) Virtual PCR prediction for screening of positively recombined RanBP9-TT KI animals. **E**) Sequence of primers used for mutant KI screening and expected size (base pair) of amplicons for *WT* and *RanBP9-TT* animals.

Figure S2. IHC detection of RanBP9 by αV5 or αHA in homozygous *RanBP9-TT* mice compared with detection by αRanBP9 specific antibody. Sections from same organs shown in Figure 2. **A**-**D**) Cerebellum; **E**-**H**) Testis; **I**-**L**) Lung. Sections from indicated organs from *RanBP9 WT* (**A**, **B**, **D**, **E**, **F**, **H**, **I**, **J**, **L**) and *RanBP9-TT* mice (**C**, **G**, **K**) were stained with αV5 (**A**, **E**, **I**), αHA (**B**, **F**, **J**) or with Hematoxylin and Eosin (H&E) (**C**, **D**, **G**, **H**, **K**, **L**). All pictures were taken with 40x objective and 10x eyepiece (400x). Scale bar = 50um.

Figure S3. IHC detection of V5 in *RanBP9-TT* mice. **A**-**O**) Selection of indicated organs/tissues from *RanBP9-TT* homozygous mice stained with αV5 specific antibody.
All pictures were taken with 40x objective and 10x eyepiece (400x). Scale bar = 50um. EXCEPT: stomach (10x = 100x, scale bar = 200um); large intestine (10x = 100x, scale bar = 200um); mammary gland (60x = 600x, scale bar = 20um).

Figure S4. IHC detection of HA in *RanBP9-TT* mice. **A-O**) Selection of indicated organs and tissues from homozygous *RanBP9-TT* mice stained with αHA specific antibody. All pictures were taken with 40x objective and 10x eyepiece (400x). Scale bar = 50um EXCEPT: stomach (10x = 100x, scale bar = 200um); large intestine (10x = 100x, scale bar = 200um); mammary gland (60x = 600x, scale bar = 20um).

Proteomic data. The datasets generated and analyzed for this study are available in the MassIVE repository [MSV000084462] at: (ftp://massive.ucsd.edu/MSV000084462/). Alternatively, they are available from corresponding author on request.

Figure S1. Generation of the RanBP9-TT Mouse by CRISPR/Cas9

A

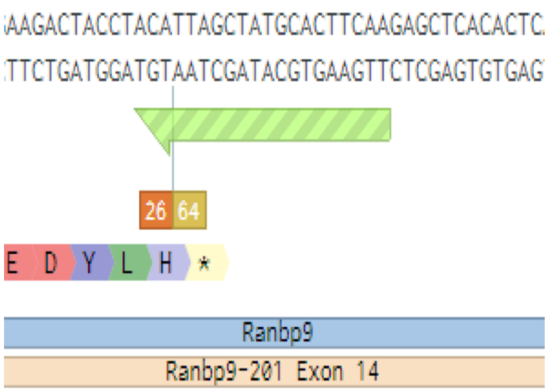

B

Sequence	PAM	On-Target Score	Off-Target Score
TTGAAGTGCATAGCTAATGT	AGG	26.2	64.2

C

Sequence	PAM	Score	Gene	Locus
TTGAAGTGCATAGCTAATGT	AGG	100.0	Ranbp9 (ENSMUSG00000038546)	chr13:+43403563
AATAAGTGCATAGCTAATGT	TAG	2.3		chr13:-29291755
TTGATGTACACAGCTAATGT	GGG	1.5		chr11:+11476826
AAAAAGTACATAGCTAATGT	TAG	1.5		chr18:+15335082
TTCATGTGCATGGCTAATGT	CAG	1.4		chr3:-63084635
TGGAAGAGCATAGCTAATGA	AGG	1.3	RP23-145F9.4 (ENSMUSG00000103083)	chr1:-171680603

D

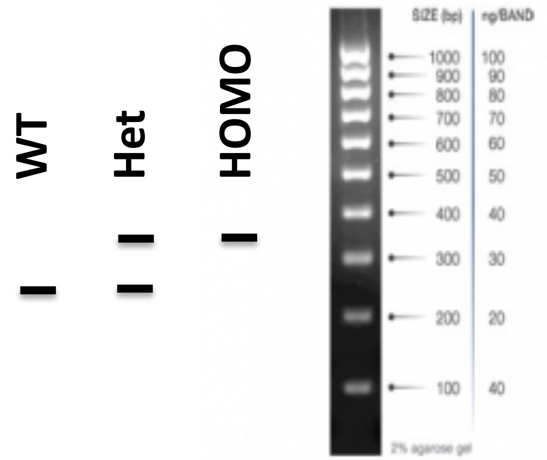

E

Forward Primer: **AAACCCACAATCTGCCAAAG**
Reverse Primer: **AAAGCGACAAAAACCTGTCC**

WT: **256 bp**
BP9-TT: 343 bp

Figure S2. IHC detection of RanBP9 by V5 in RanBP9-TT mice.

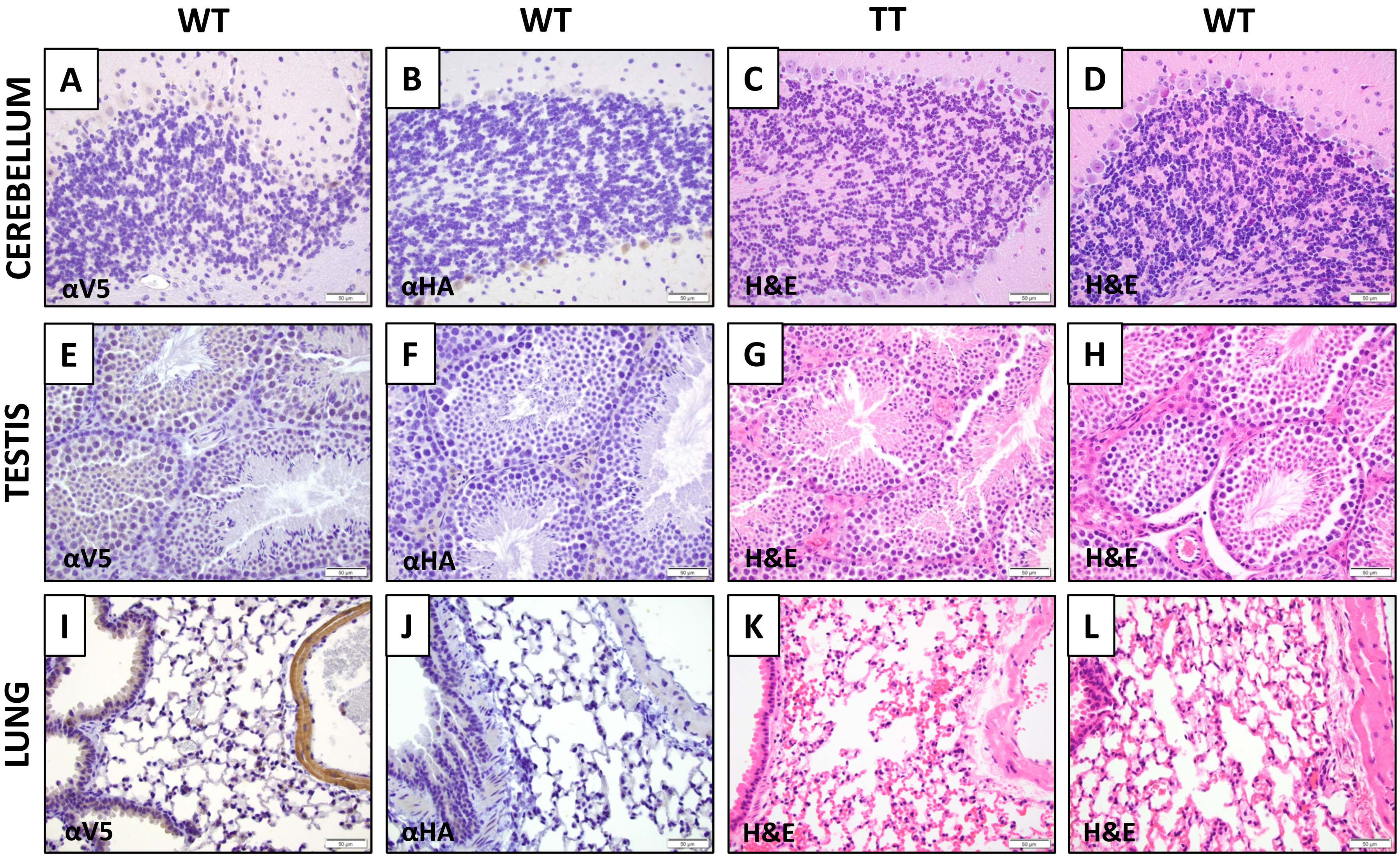

Figure S3. IHC detection of RanBP9 by V5 in RanBP9-TT mice.

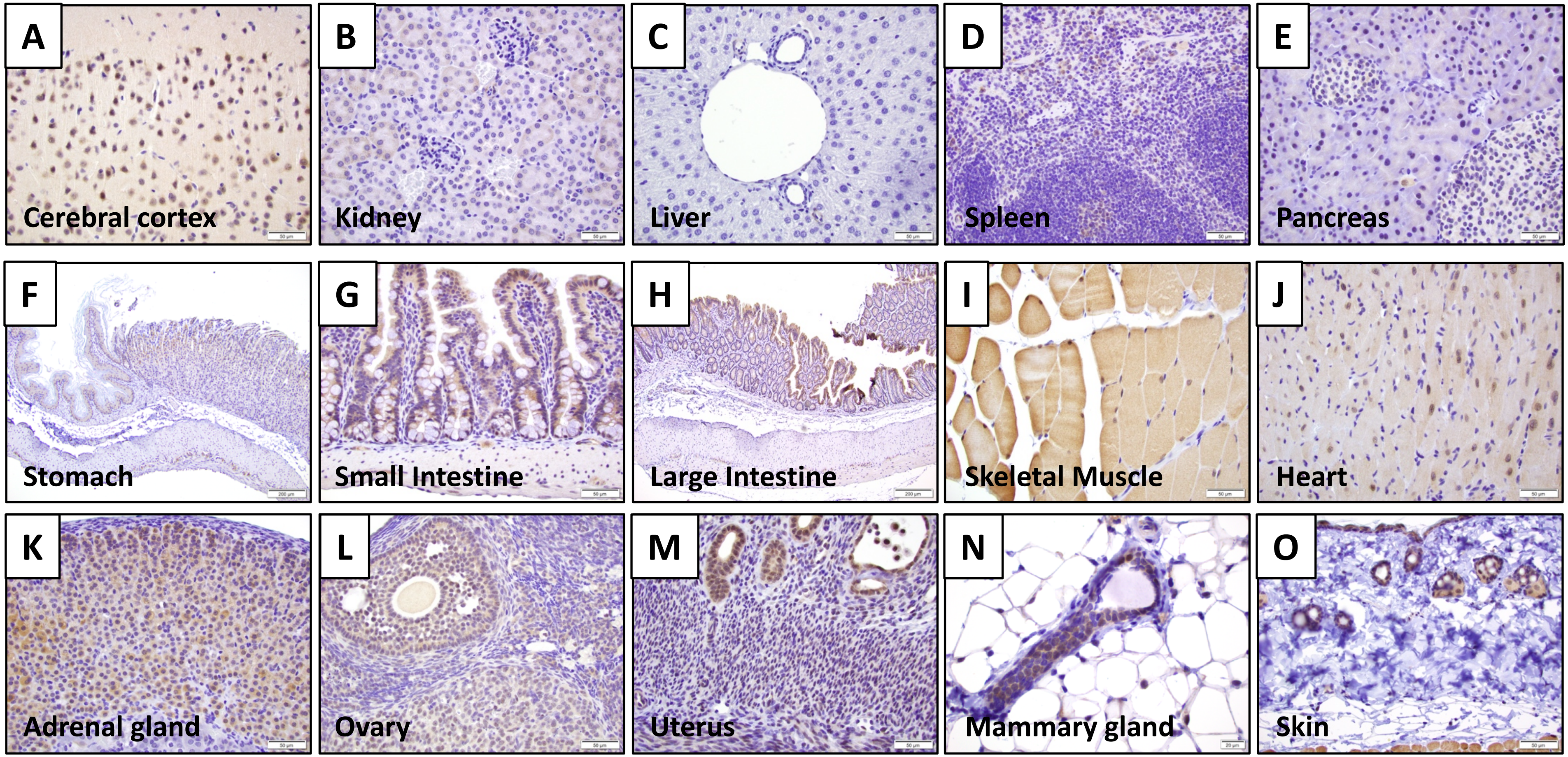

Figure S4. IHC detection of RanBP9 by HA in RanBP9-TT mice.

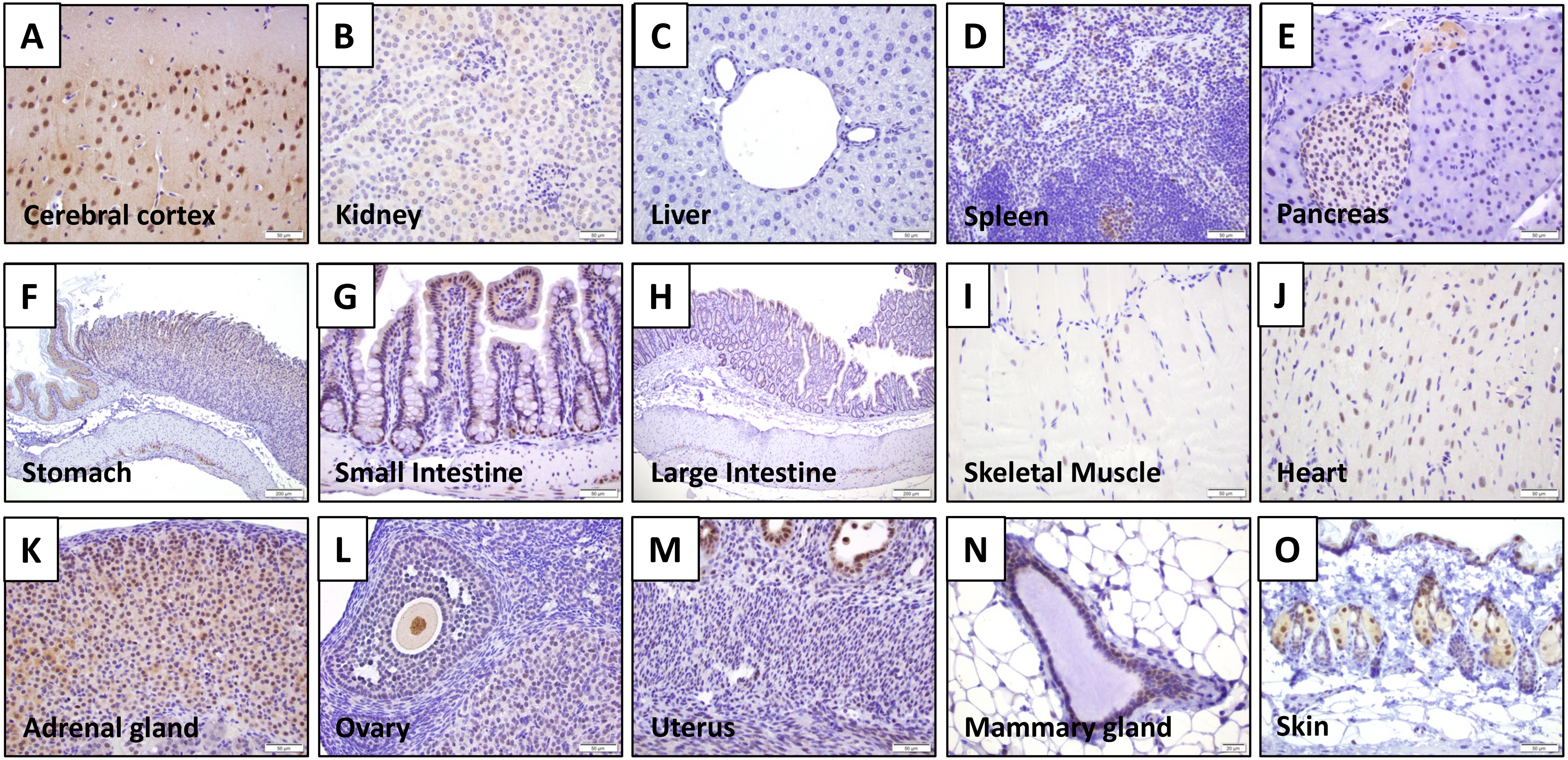

Supplementary Table 1. Primary antibodies used for this investigation

	Name of the antibody	Company website/Validation data	Dilution			Amount
			WB		IHC	IP
1	rabbit aRanBP9 antibody, cat. nr. HPA050007	https://www.proteinatlas.org/ENSG00000010017-RANBP9/antibody#western_blot<br>We have previously used and validate this antibody with RANBP9 KO cells<br>(Palmieri et al., 2016; Tessari et al., 2018)	-----	-----	1:50	-----
2	goat V5 antibody, Abcam cat. nr. ab95038	https://www.abcam.com/v5-tag-antibody-ab95038.html	-----	-----	1:350	-----
3	rabbit HA, Cell Signaling cat. nr. C29F4	https://www.cellsignal.com/products/primary-antibodies/ha-tag-c29f4-rabbit-mab/3724	-----	-----	1:800	-----
4	RANBP9, Abcam cat. nr. ab140627	https://www.abcam.com/ranbp9-antibody-epr9920b-ab140627.html	1:2,000	5% milk	-----	-----
5	GAPDH, Cell Signaling cat. nr. 3683S	https://www.cellsignal.com/products/antibody-conjugates/gapdh-14c10-rabbit-mab-hrp-conjugate/3683	1:3,000	5% milk	-----	-----
6	monoclonal HA-Agarose antibody, Sigma cat. nr. A2095-1ML	https://www.sigmaaldrich.com/catalog/product/sigma/a2095?lang=en&region=US	-----	-----	-----	5ug
7	RMND5A, Novus Biologicals cat. nr. NBP1-92337	https://www.novusbio.com/products/rmnd5a-antibody_nbp1-92337	1:1,000	5% milk	-----	-----
8	WDR26, Abcam cat. nr. ab85962	https://www.abcam.com/wdr26-antibody-ab85962.html	1:1,000	5% milk	-----	-----
9	ARMC8, Proteintech cat. nr. 12653-1-AP	https://www.ptglab.com/products/ARMC8-Antibody-12653-1-AP.htm	1:1,000	5% milk	-----	-----
10	Muskelin, Proteintech cat. nr. 14735-1-AP	https://www.ptglab.com/products/MKLN1-Antibody-14735-1-AP.htm	1:1,000	5% milk	-----	-----
11	C20orf11 (a.k.a. GID8), Proteintech cat. nr. 24479-1-AP	https://www.ptglab.com/products/C20orf11-Antibody-24479-1-AP.htm	1:1,000	5% milk	-----	-----
12	MAEA, R&D Systems cat. nr. AF7288	https://www.rndsystems.com/products/human-mouse-rat-emp-maea-antibody_af7288	1:2,000	5% milk	-----	-----
13	RanBP10, Millipore SIGMA; cat. nr. SAB3500163	https://www.sigmaaldrich.com/catalog/product/sigma/sab3500163?lang=en&region=US	1:1,000	5% milk	-----	-----
14	Nucleolin, D4C7O; Cell Signaling; rabbit mAB cat. nr. 14574	https://www.cellsignal.com/products/primary-antibodies/nucleolin-d4c7o-rabbit-mab/14574	1:2,000	5% milk	-----	-----
15	V5 antibody, Invitrogen cat. nr. R96025	https://www.thermofisher.com/antibody/primary/target/v5%20tag	1:1,000	5% milk	-----	5ug
15	mouse Vinculin antibody, Abcam cat. nr. Ab130007	https://www.abcam.com/vinculin-antibody-vin-54-ab130007.html	1:2,000	5% milk	-----	-----

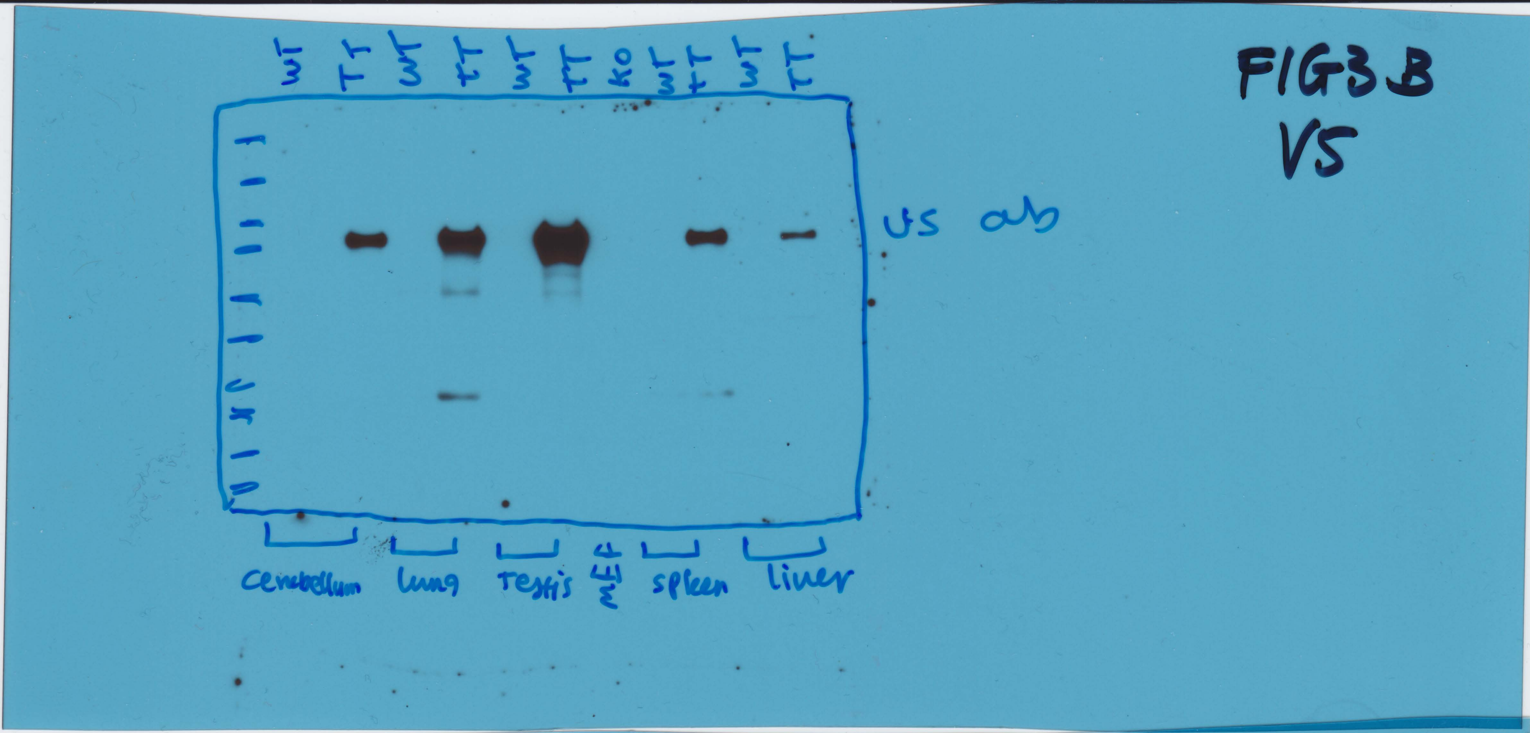
FIG3B
V5
WT TT WT TT WT TT KO WT TT WT TT
V5 ab
cerebellum
lung
testis
MEF
spleen
liver

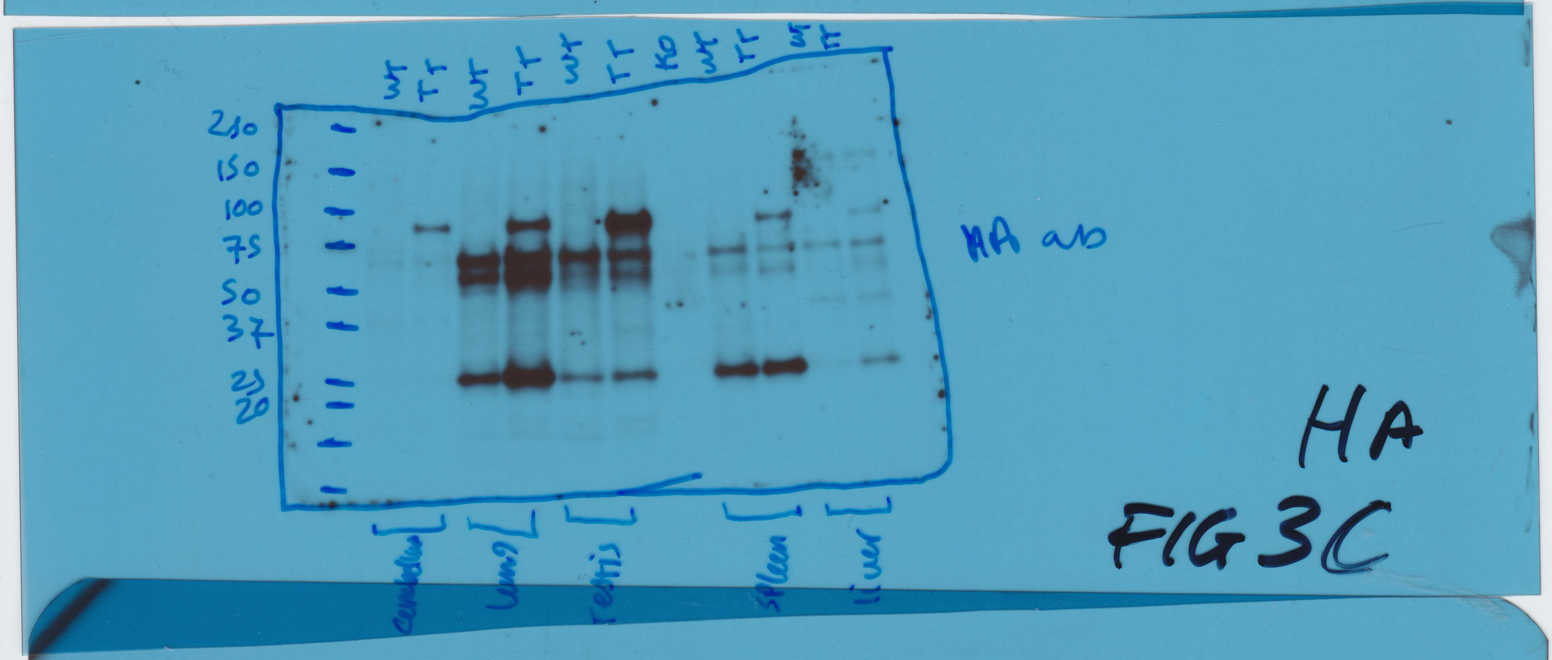
WT TT WT TT WT TT KO WT TT WT TT
250
150
100
75
50
37
25
20
HA ab
HA
FIG3C
cerebellum
lung
testis
spleen
liver

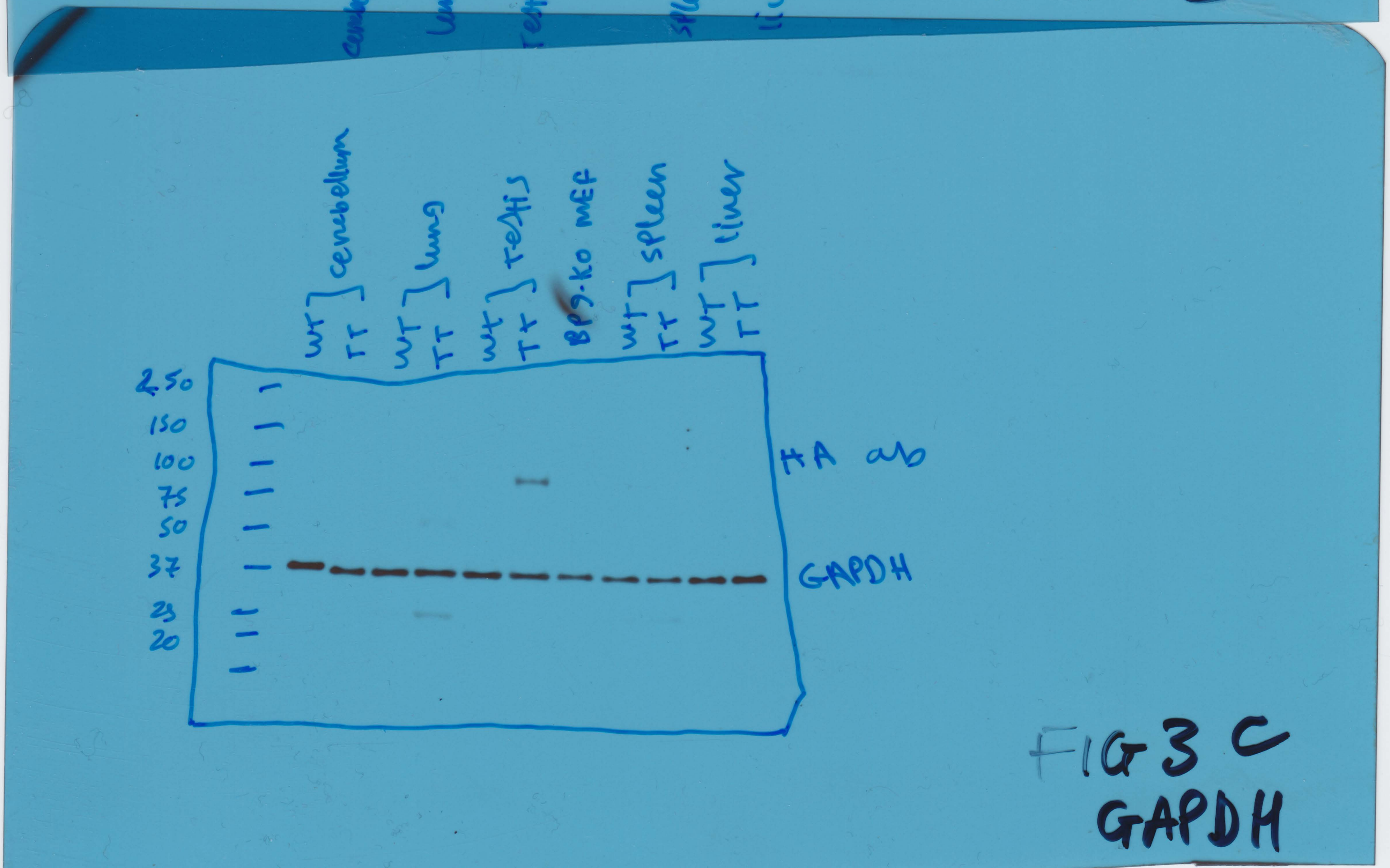
cerebellum
lung
testis
B29-KO MEF
spleen
liver
WT TT WT TT WT TT WT TT WT TT
250
150
100
75
50
37
25
20
HA ab
GAPDH
FIG3C
GAPDH

FIG3 A
GAPDH

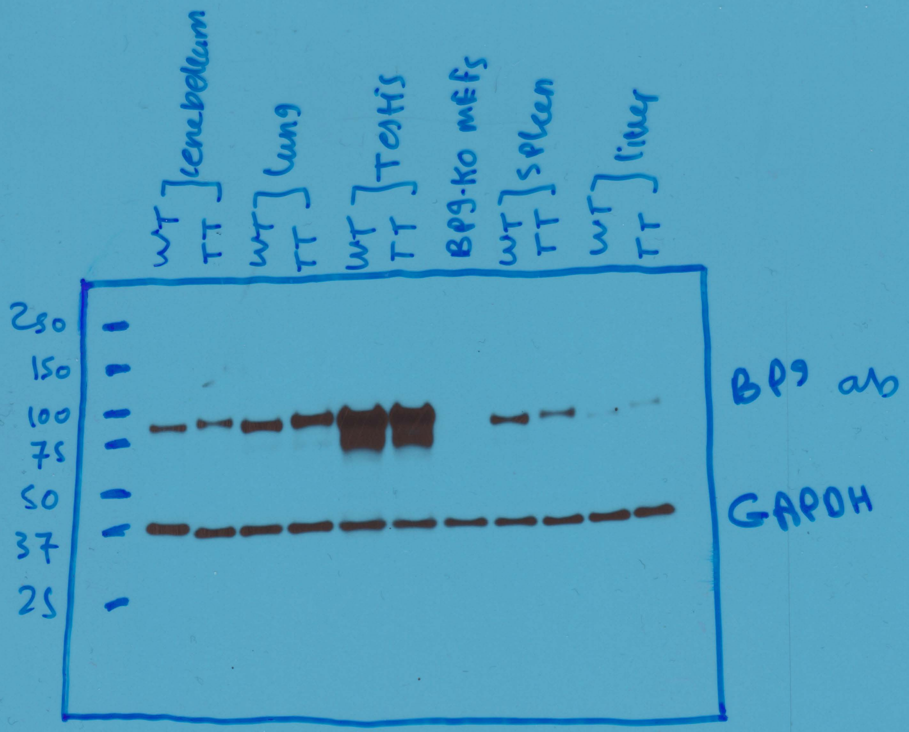

FIG3B
GAPDH

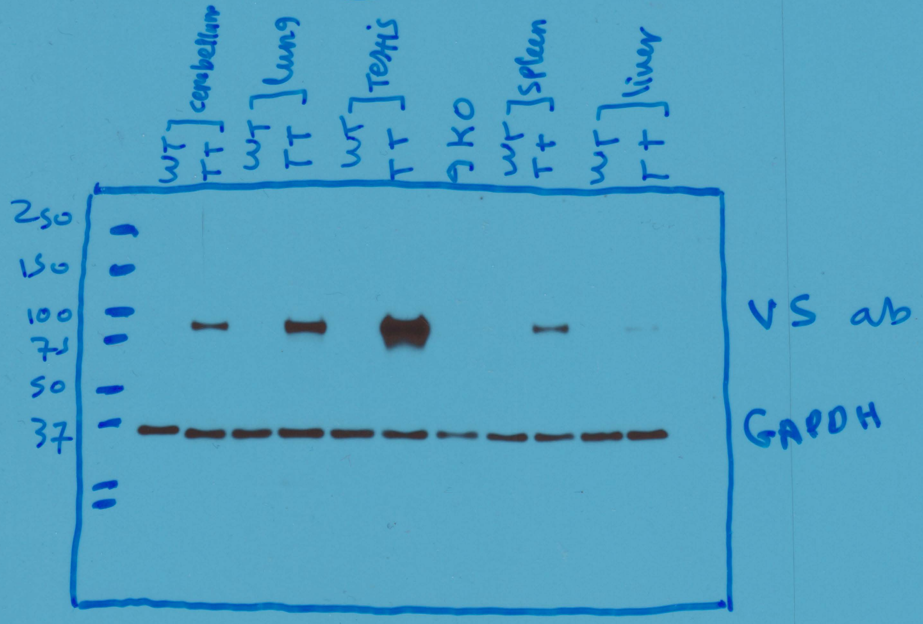

FIG 4A EN20 1.16.20

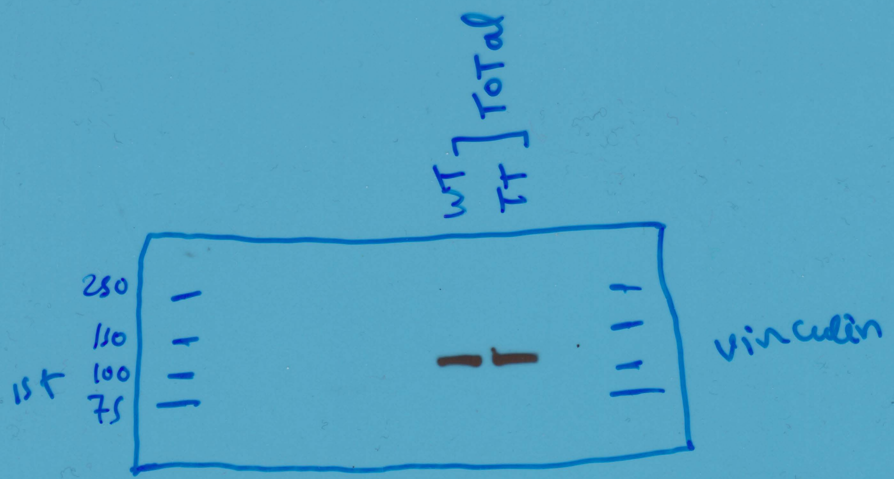

FIG 4A EN20 1.16.20

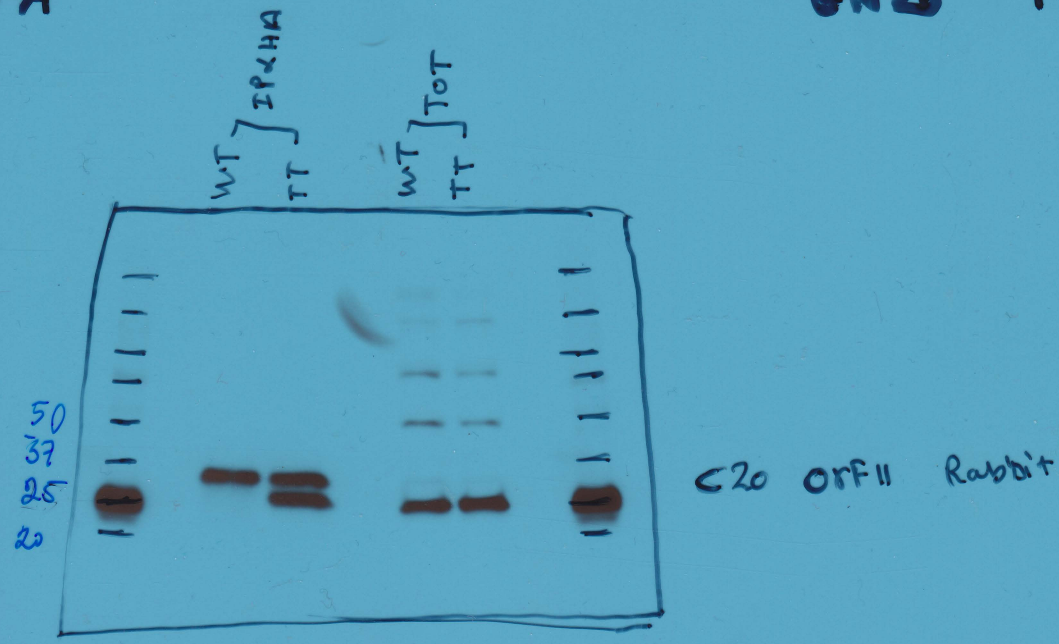

FIG 4A

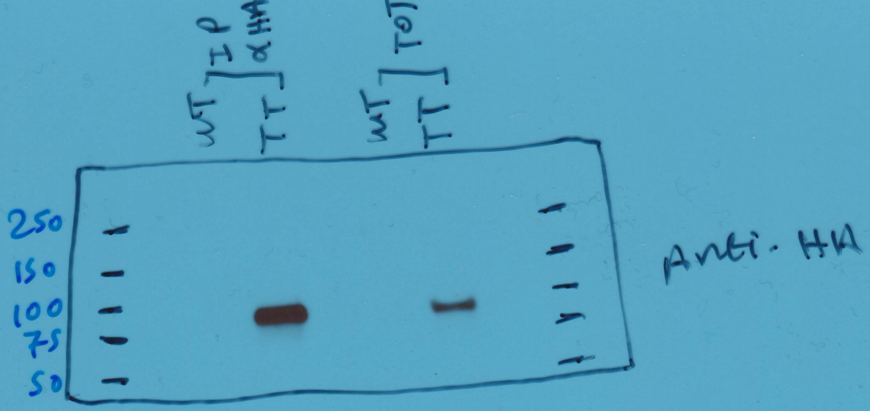

ENZO 1-16-20

FIG 4A

ENZO 1-16-20

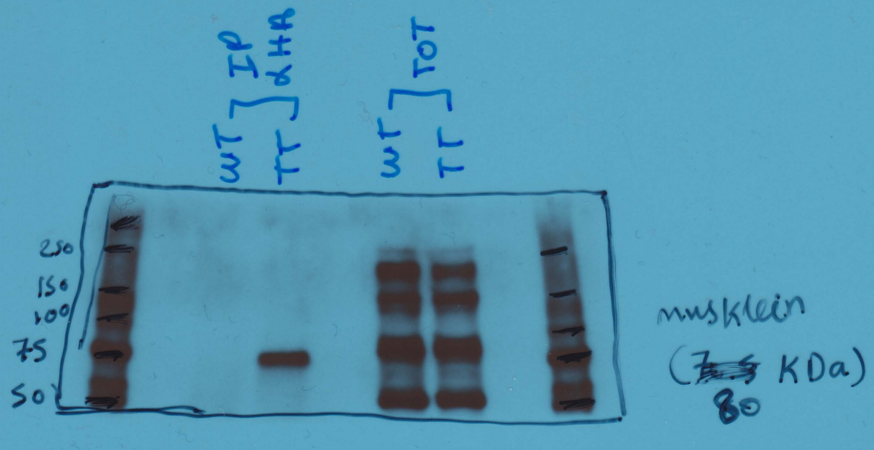

ENRO 1·16·20

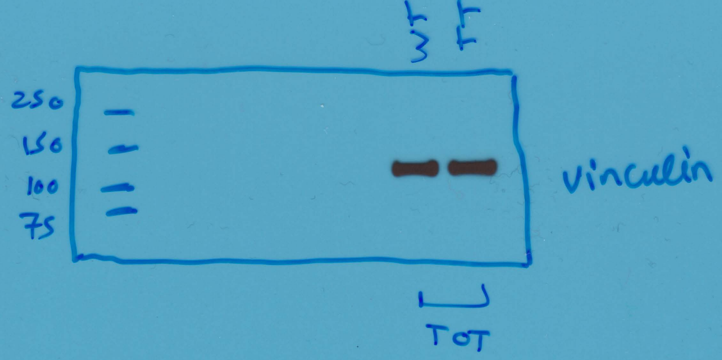

FIG 4B

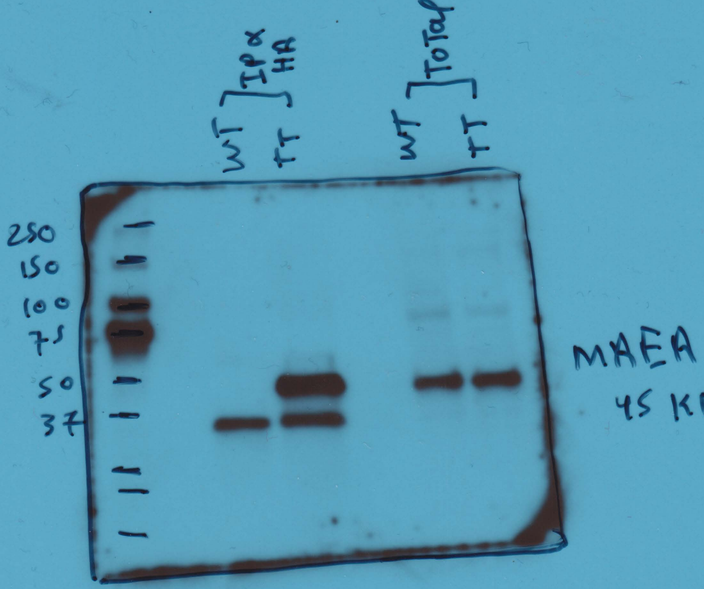

ENRO 1·16·20

FIG 4B

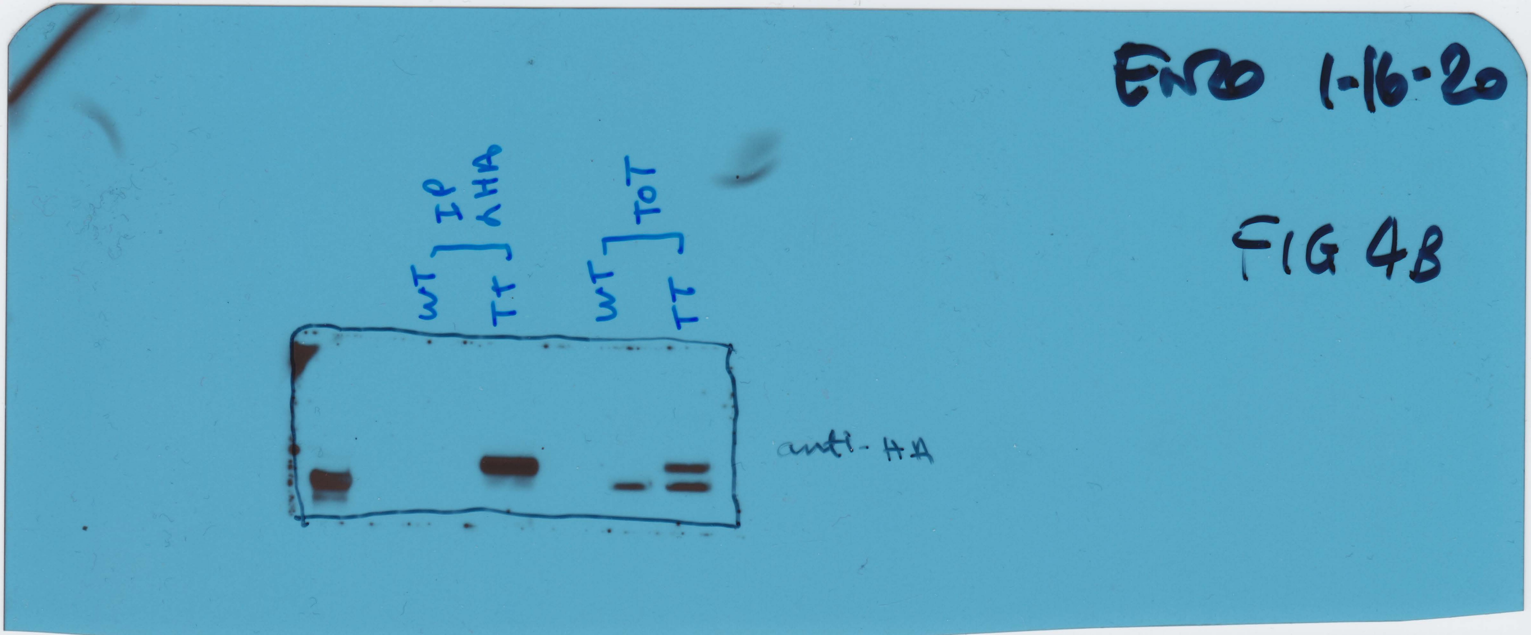
ENZO 1-16-20
FIG 4B
IP αHA
ToT
WT
TT
WT
TT
anti-HA

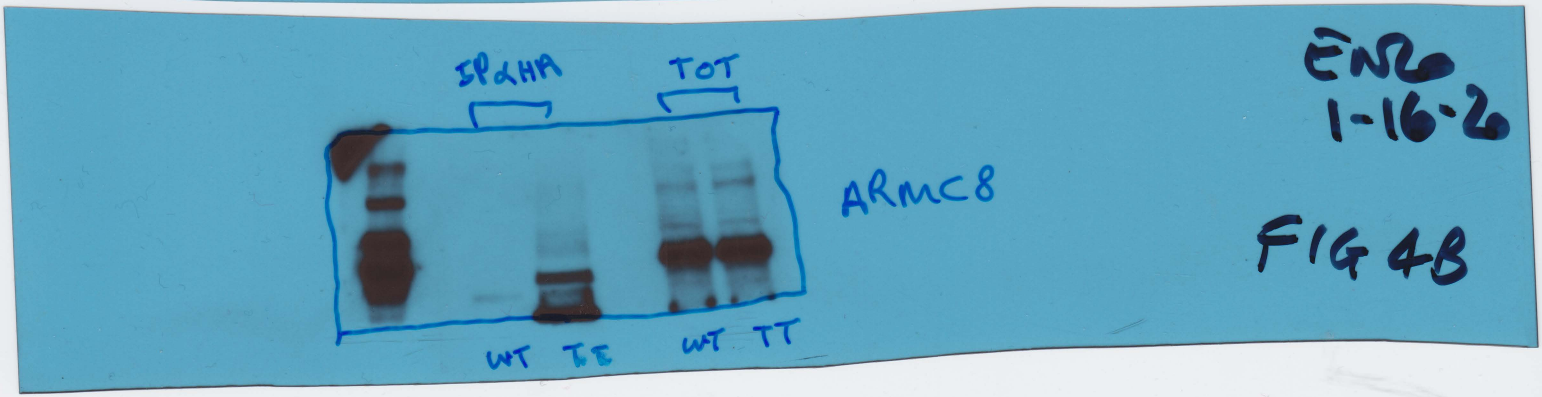
ENZO 1-16-20
IPαHA
TOT
ARMC8
FIG 4B
WT
TT
WT
TT

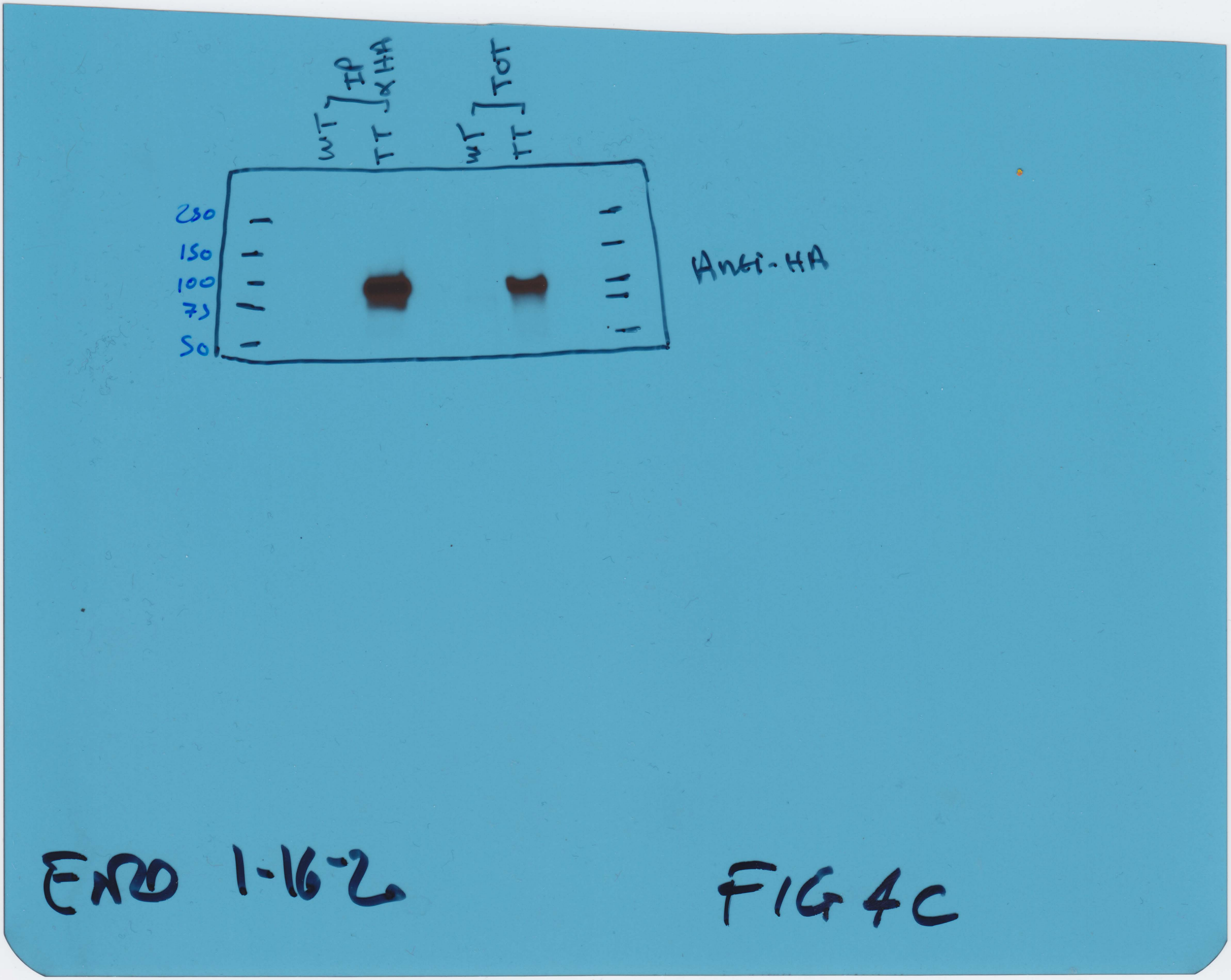
WT
TT
IP αHA
WT
TT
TOT
250
150
100
75
50
Anti-HA
EMD 1-16-20
FIG 4c

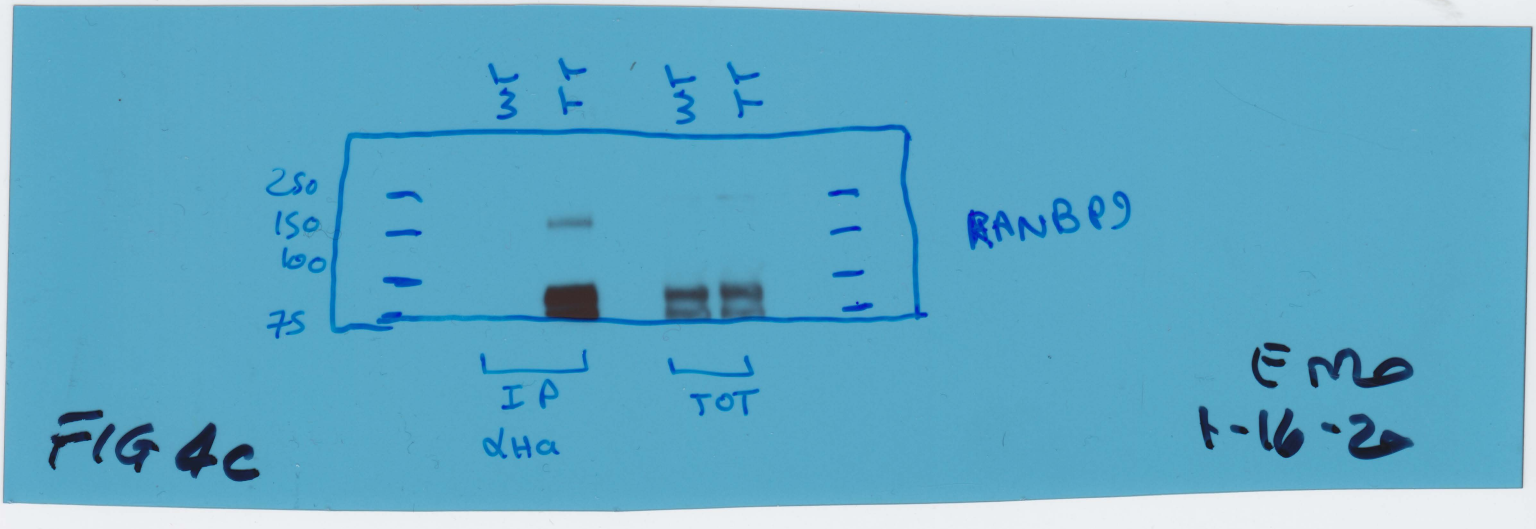
WT
TT
WT
TT
250
150
100
75
RANBP9
IP
αHa
TOT
FIG 4c
EMD
1-16-20

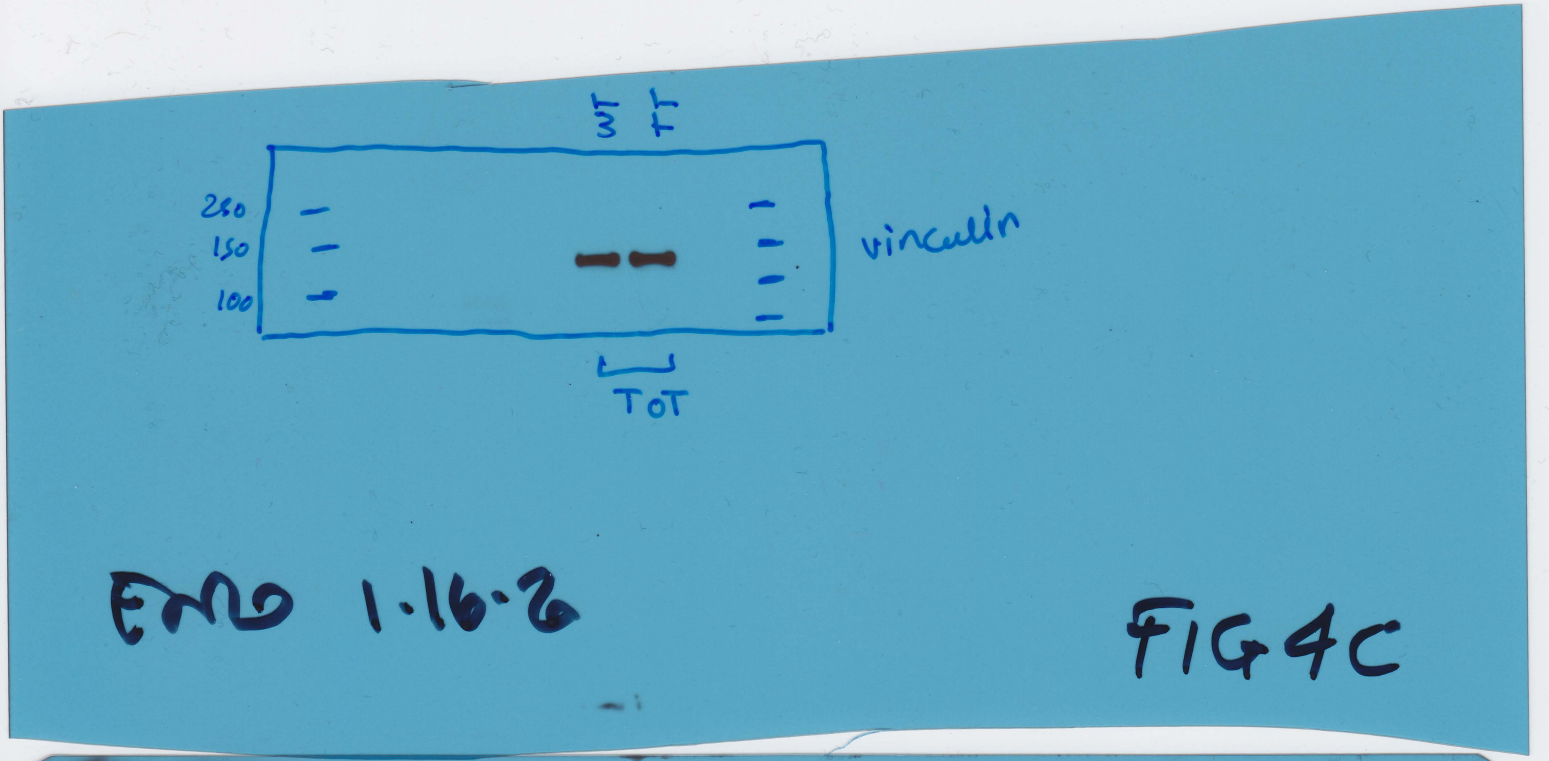
WT
TT
250
150
100
vinculin
TOT
ENRO 1.16.2
FIG4C

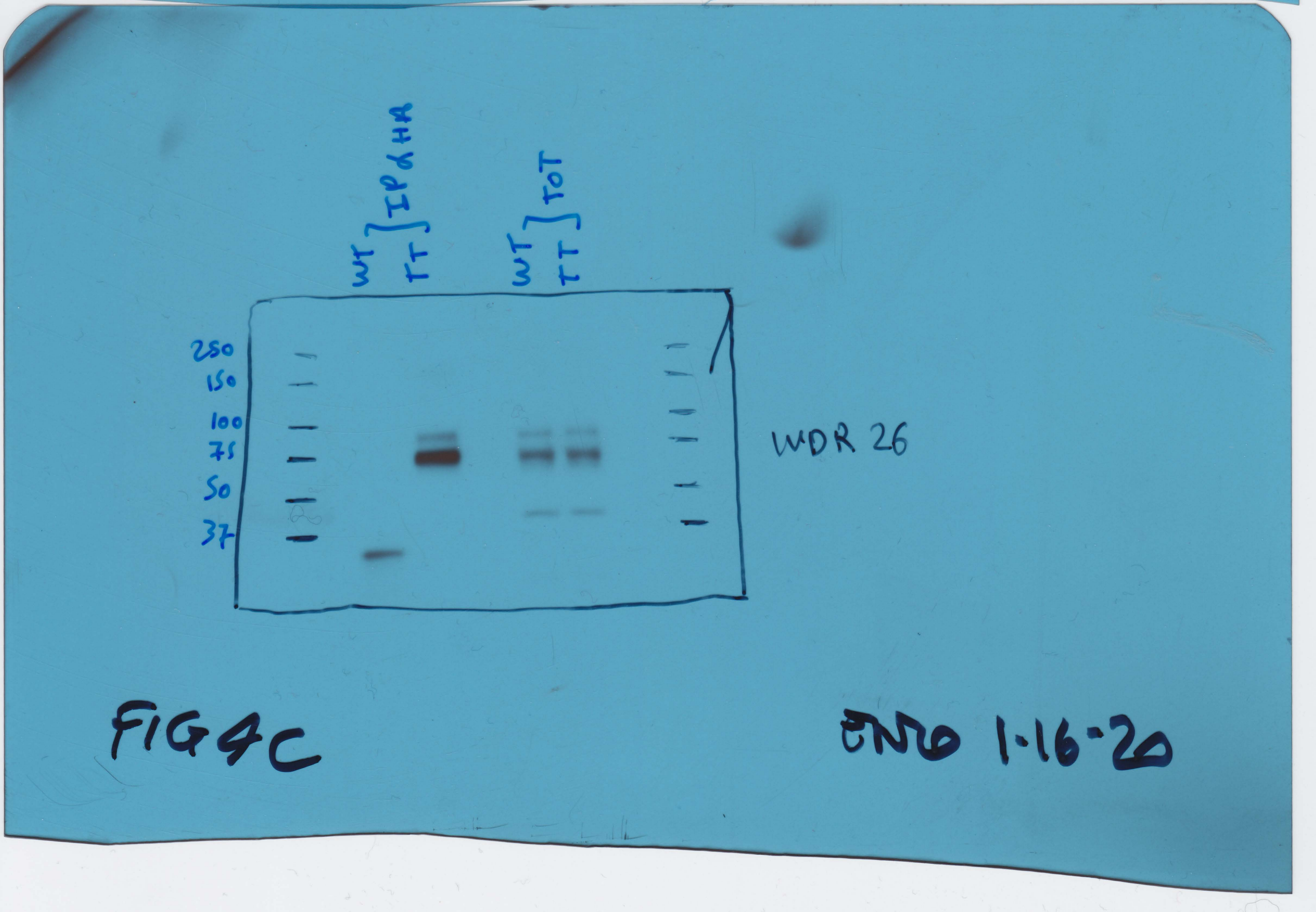
WT
TT
IP αHA
WT
TT
TOT
250
150
100
75
50
37
WDR 26
FIG4C
ENRO 1.16.20

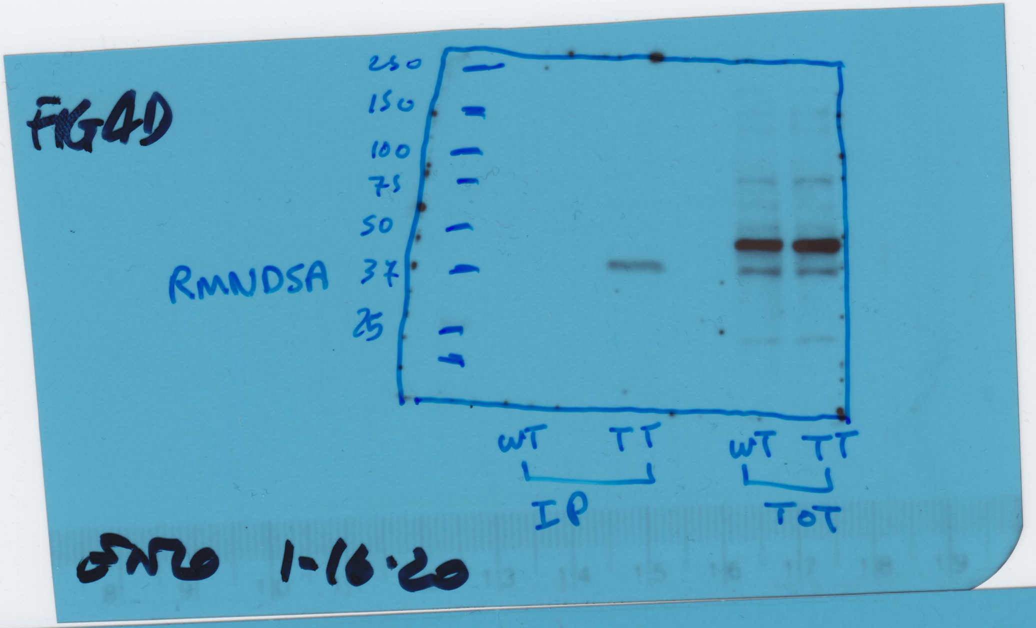
FIG 4D
250
150
100
75
50
37
25
RMND5A
WT
TT
IP
WT
TT
TOT
ENZO 1-16-20

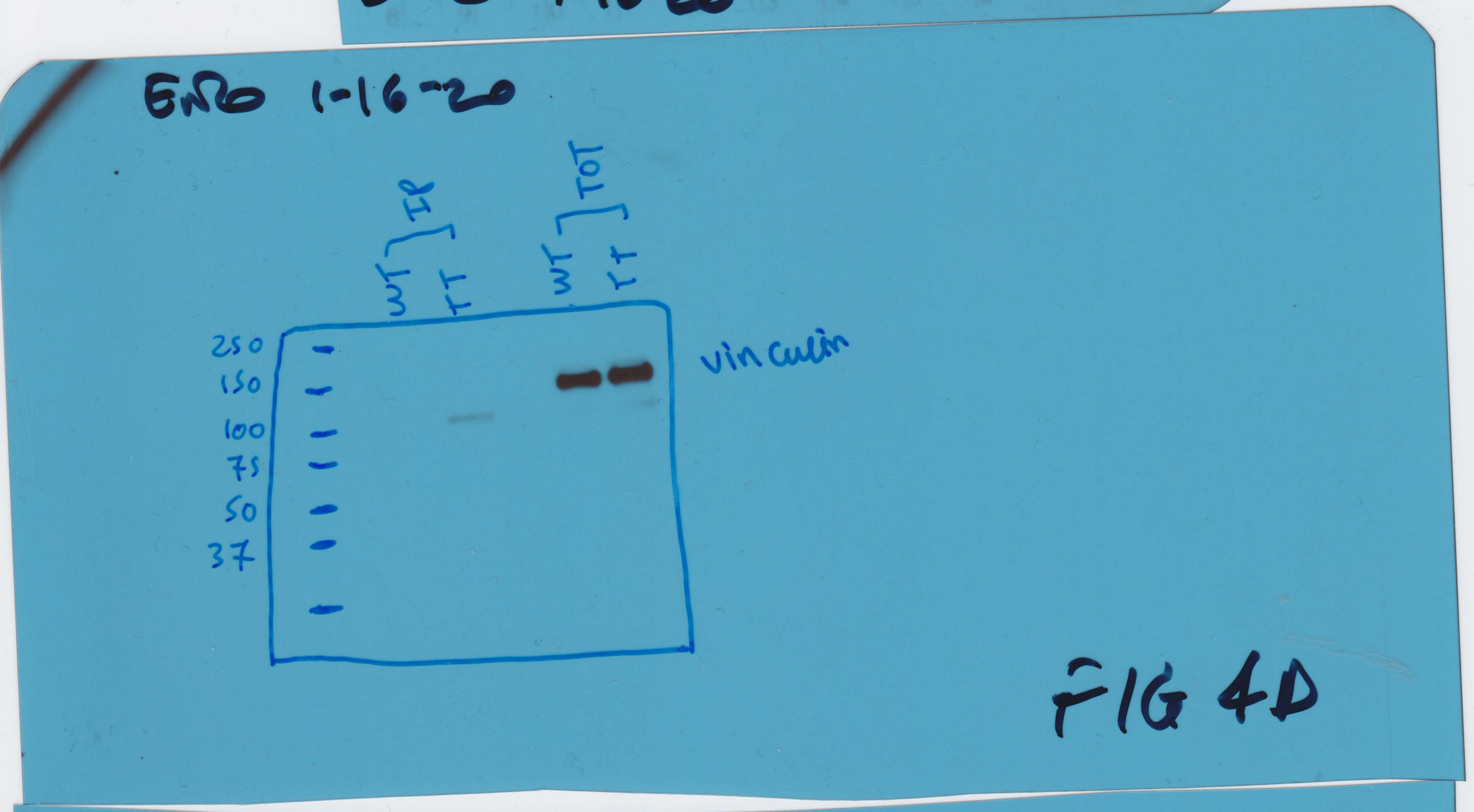
ENZO 1-16-20
WT
TT
IP
WT
TT
TOT
250
150
100
75
50
37
vinculin
FIG 4D

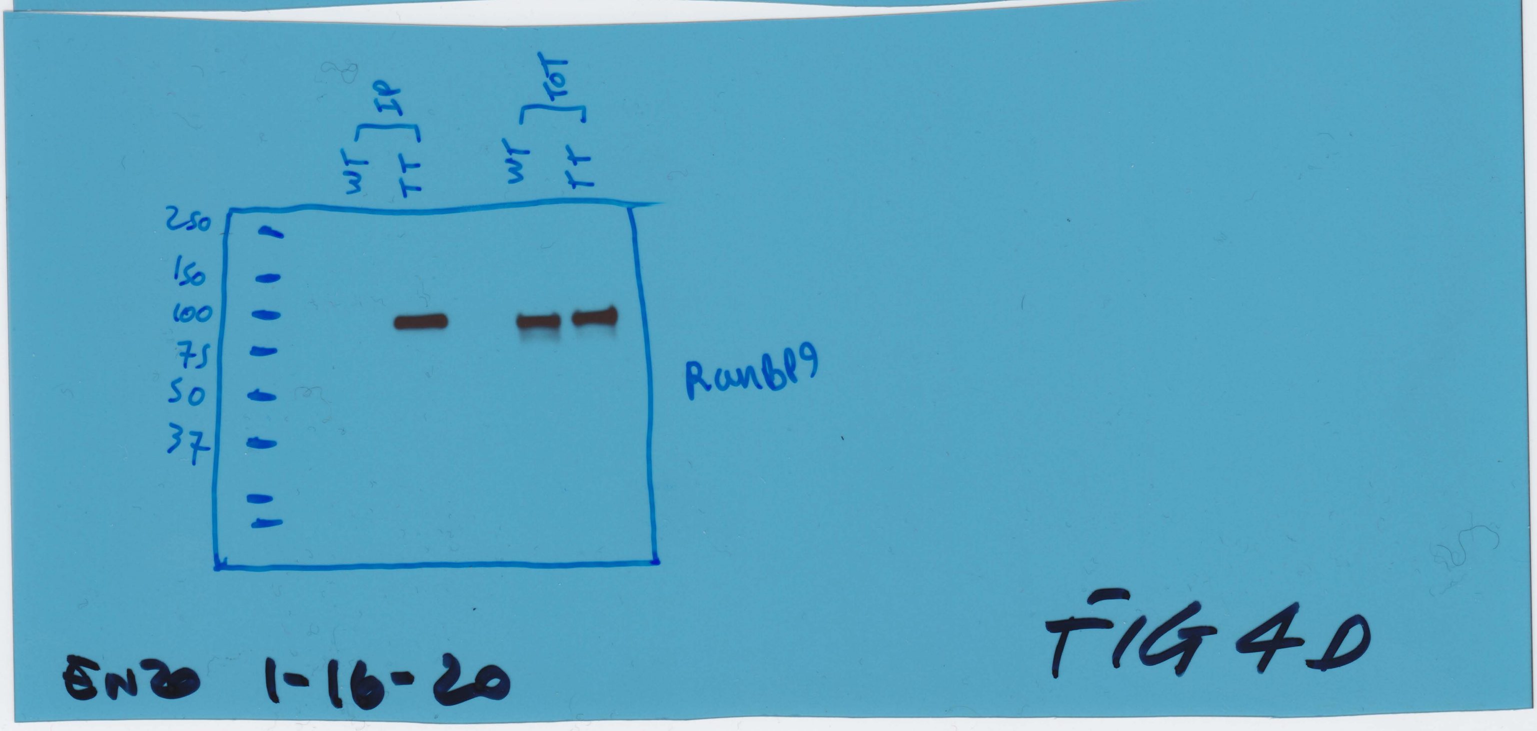
WT
TT
IP
WT
TT
TOT
250
150
100
75
50
37
Ranbp9
FIG 4D
ENZO 1-16-20

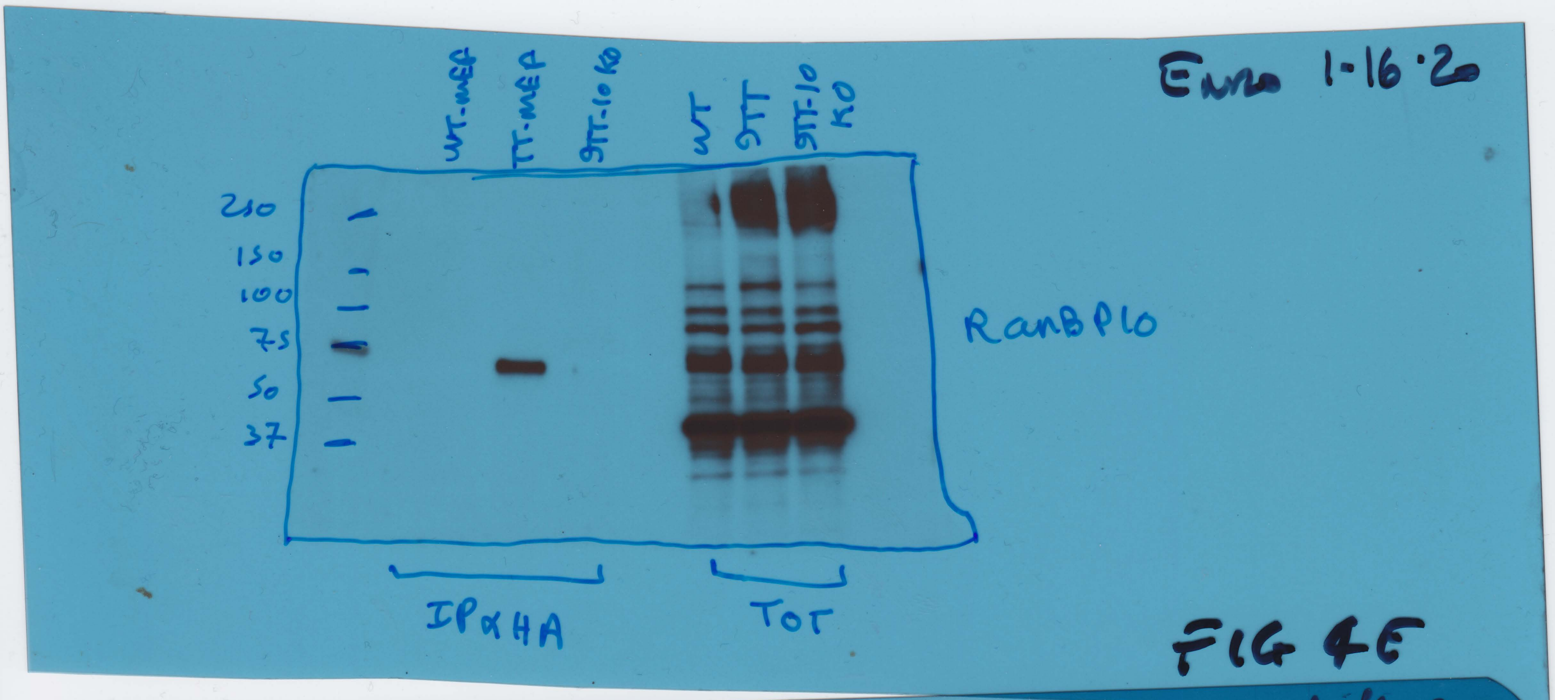
Enzo 1·16·20
WT-MEF
TT-MEF
9TT-10 KO
WT
9TT
9TT-10 KO
250
150
100
75
50
37
RanBP10
IP αHA
TOT
FIG 4E

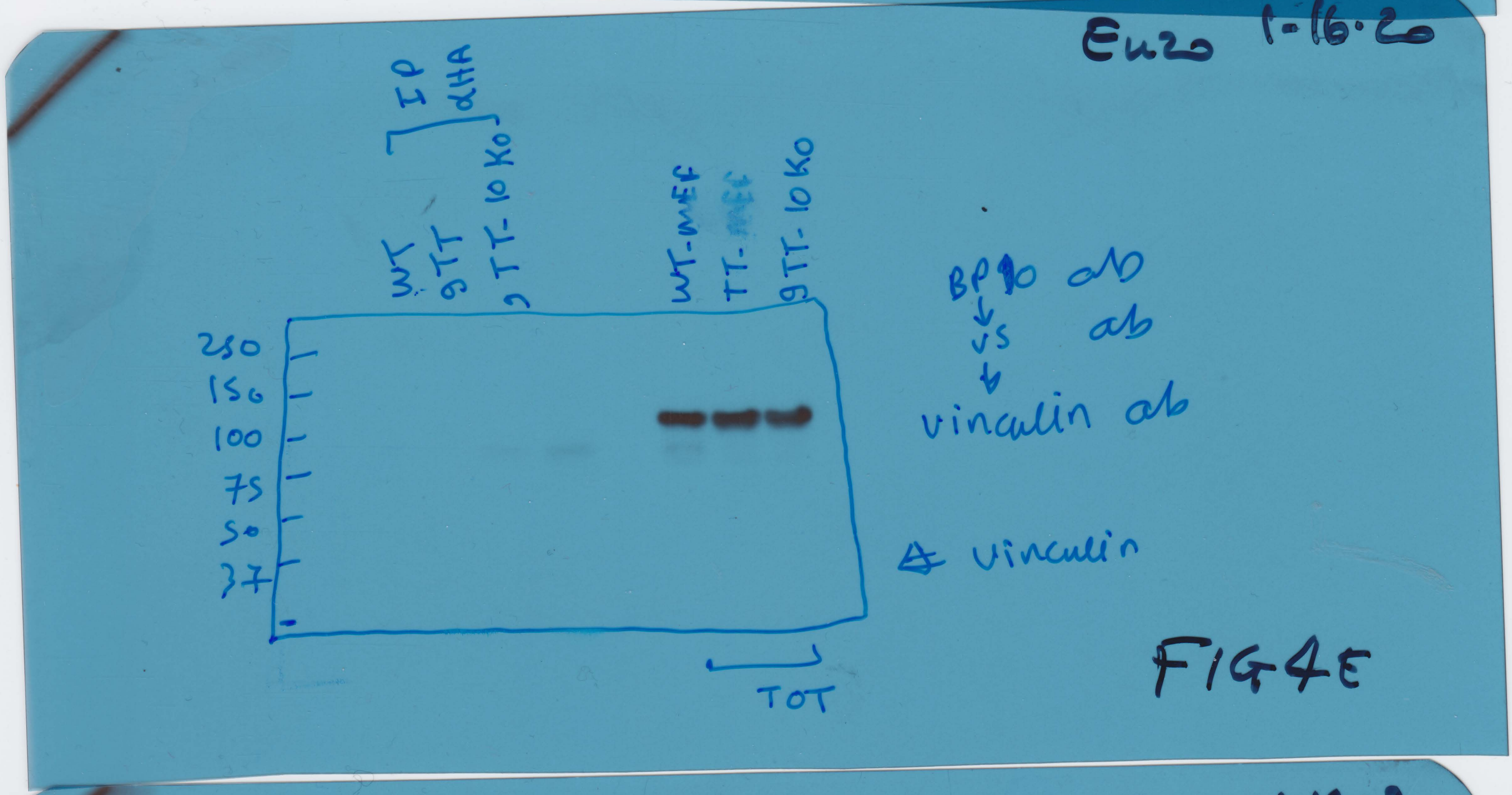
Enzo 1·16·20
IP αHA
WT
9TT
9TT-10 KO
WT-MEF
TT-MEF
9TT-10 KO
250
150
100
75
50
37
BP10 ab
vs ab
vinculin ab
vinculin
TOT
FIG 4E

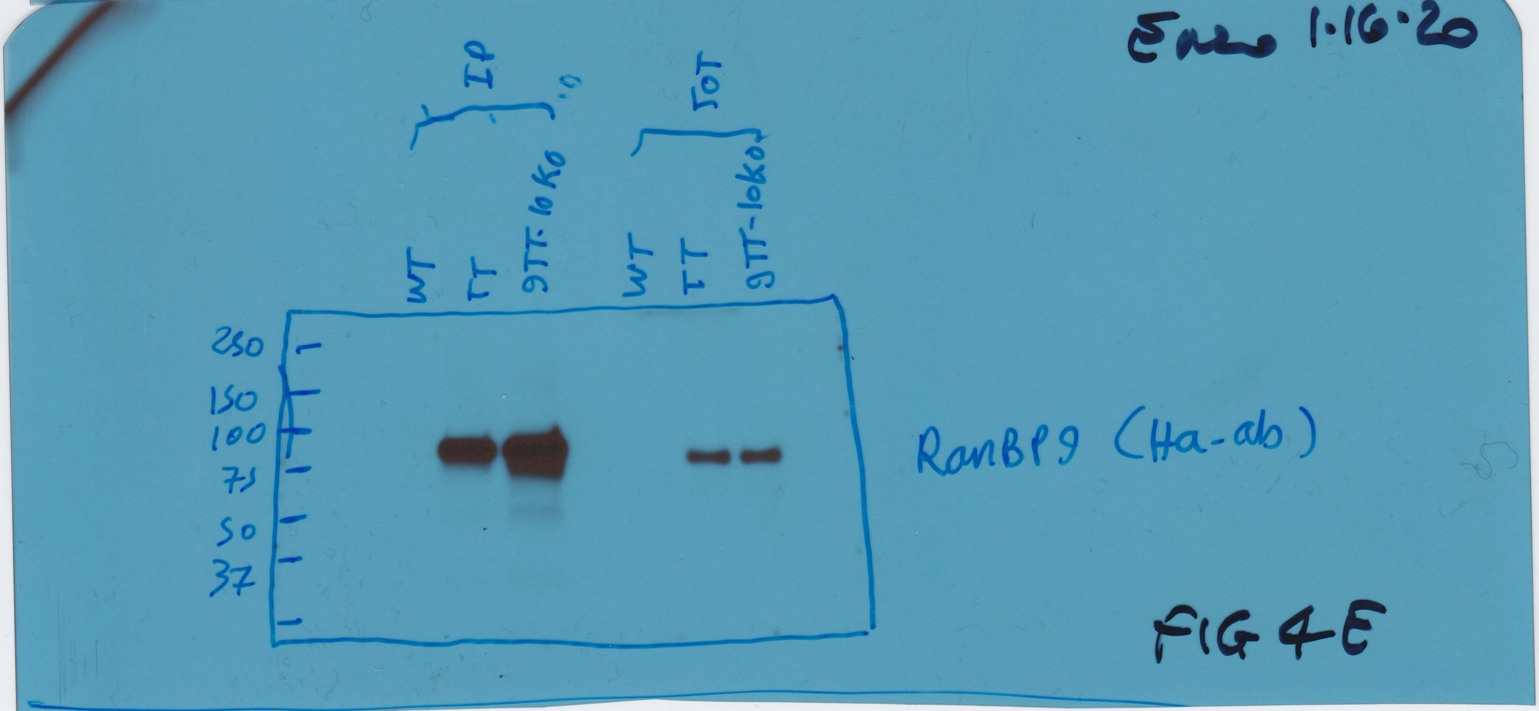
Enzo 1·16·20
IP
TOT
WT
TT
9TT-10 KO
WT
TT
9TT-10 KO
250
150
100
75
50
37
RanBP9 (Ha-ab)
FIG 4E

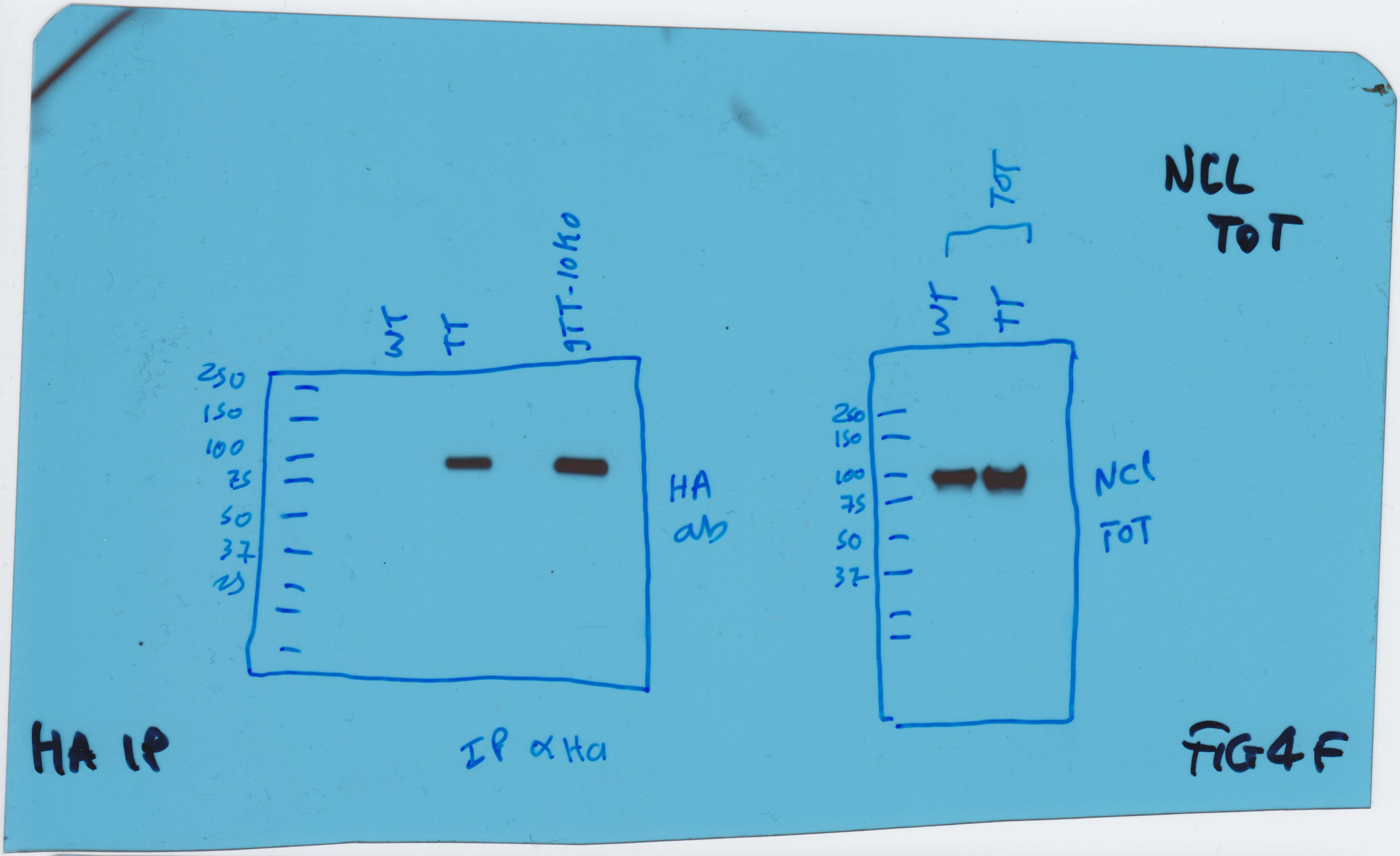
NCL TOT
WT
TT
9TT-10KO
250
150
100
75
50
37
25
HA ab
TOT
WT
TT
NCl
TOT
HA IP
IP αHa
FIG4F

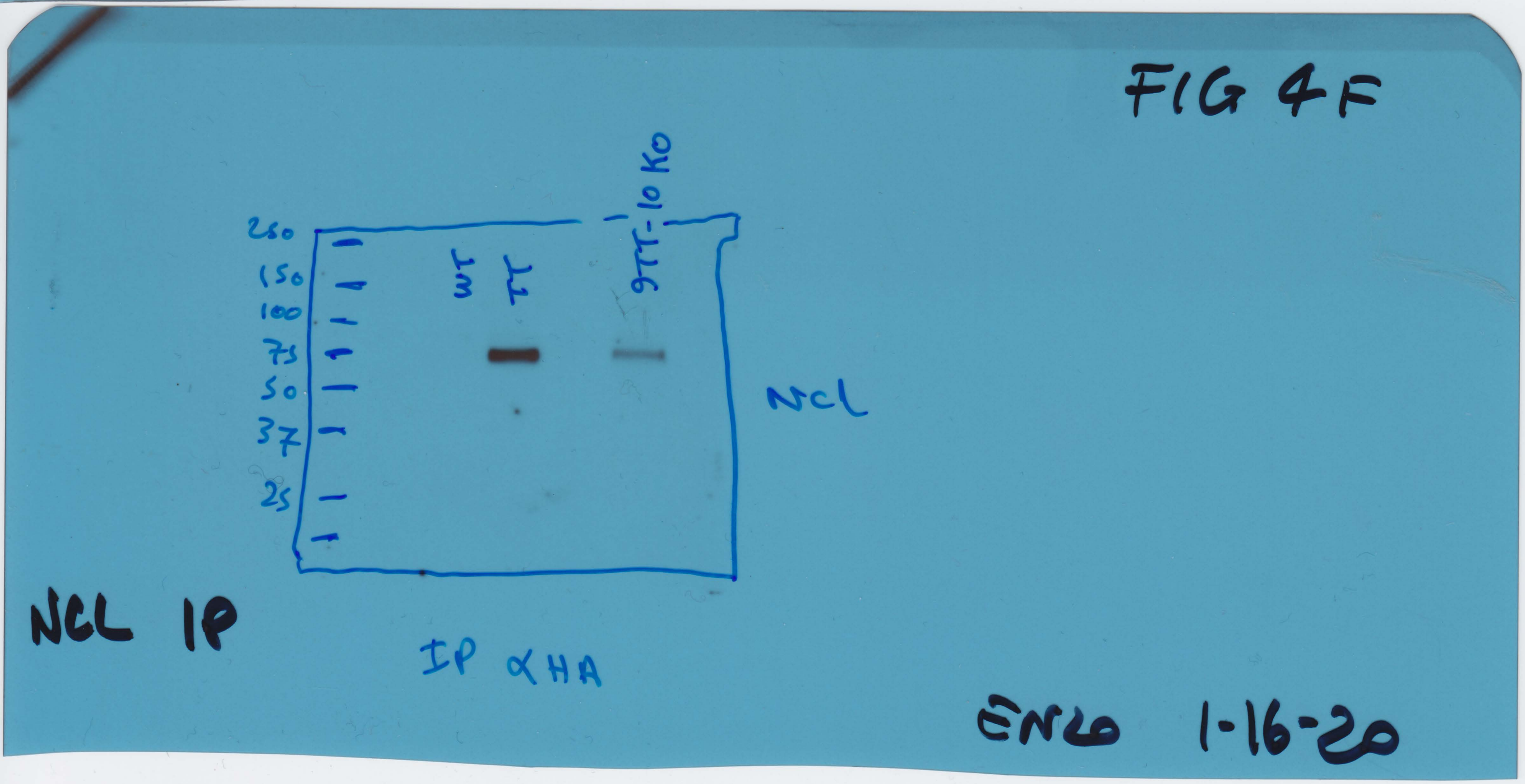
FIG 4F
WT
TT
9TT-10KO
250
150
100
75
50
37
25
Ncl
NCL IP
IP αHA
1-16-20

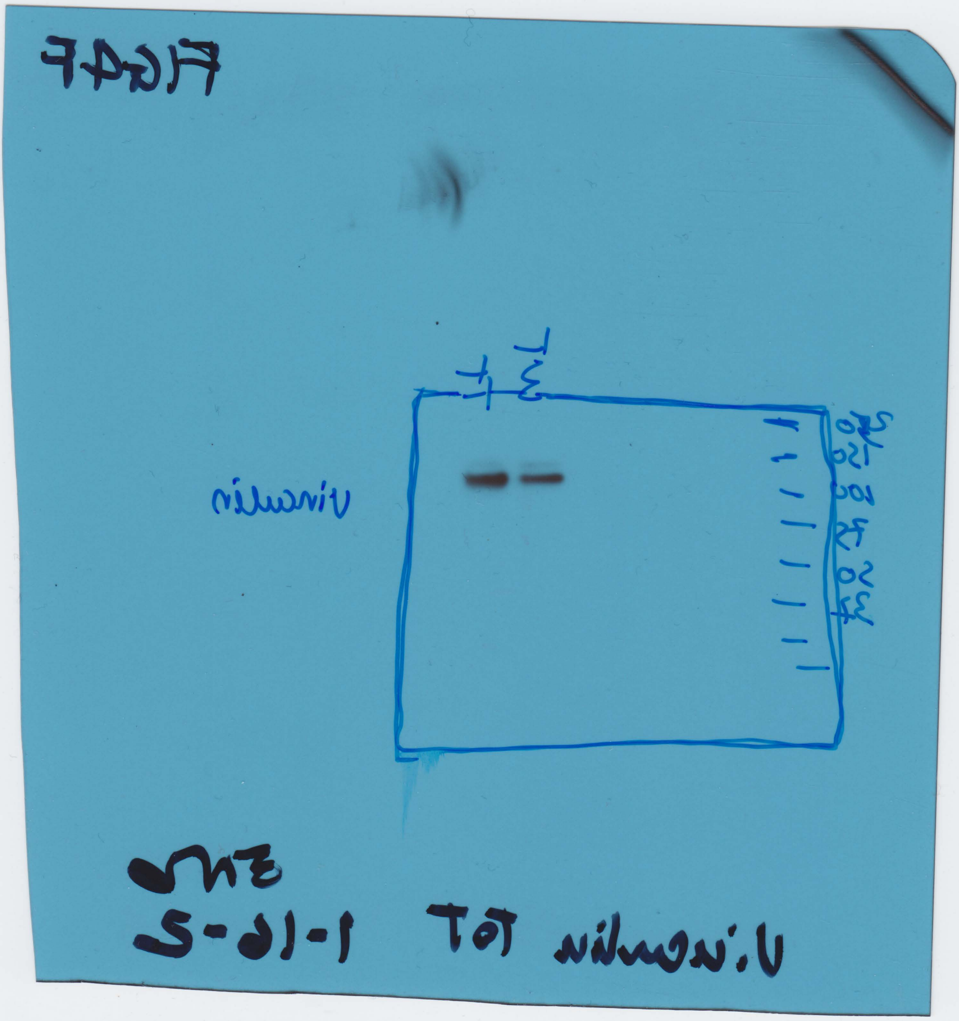
FIG 4F
TT
WT
250
150
100
75
50
37
25
vinculin
ENZ
1-16-20 TOT vinculin

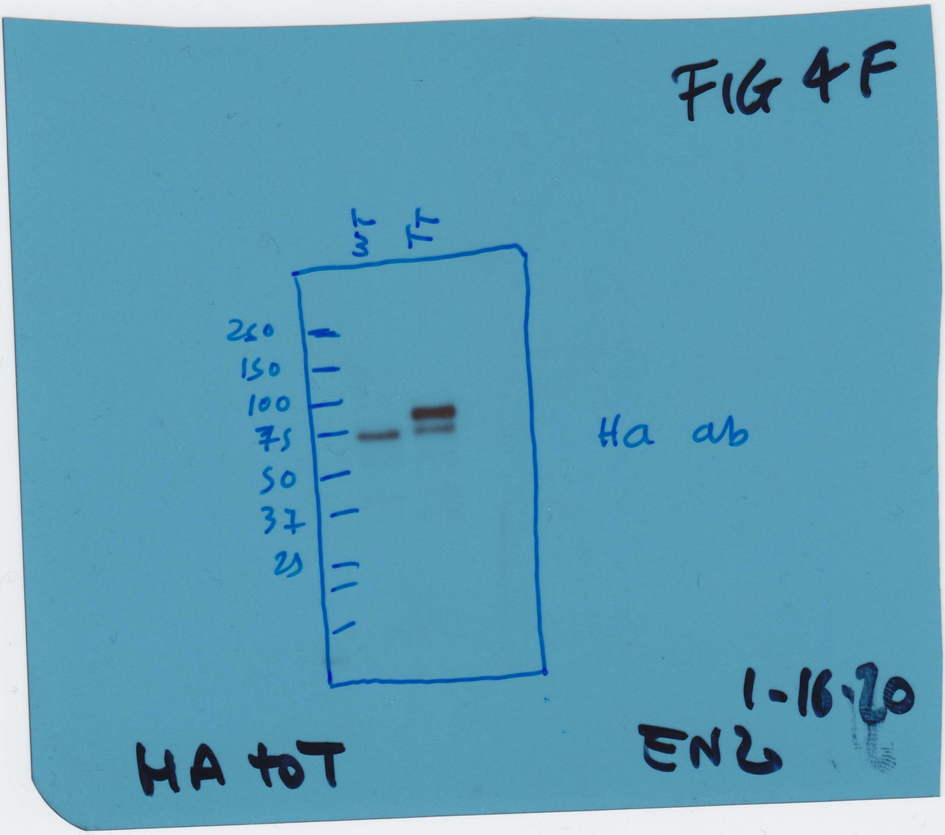
FIG 4F
WT
TT
250
150
100
75
50
37
25
Ha ab
1-16-20
HA tot
ENZ
